# Antiviral and Anti‐Inflammatory Treatment with Multifunctional Alveolar Macrophage‐Like Nanoparticles in a Surrogate Mouse Model of COVID‐19

**DOI:** 10.1002/advs.202003556

**Published:** 2021-05-12

**Authors:** Bin Li, Wei Wang, Weifeng Song, Zheng Zhao, Qingqin Tan, Zhaoyan Zhao, Lantian Tang, Tianchuan Zhu, Jialing Yin, Jun Bai, Xin Dong, Siyi Tan, Qunying Hu, Ben Zhong Tang, Xi Huang

**Affiliations:** ^1^ Center for Infection and Immunity Guangdong Provincial Key Laboratory of Biomedical Imaging The Fifth Affiliated Hospital of Sun Yat‐sen University Zhuhai Guangdong 519000 China; ^2^ Southern Marine Science and Engineering Guangdong Laboratory Zhuhai Guangdong 519000 China; ^3^ Engineering Research Center of Tibetan Medicine Detection Technology, Ministry of Education Xizang Minzu University Xianyang Shaanxi 712082 China; ^4^ Department of Chemistry The Hong Kong University of Science and Technology Clear Water Bay Kowloon Hong Kong 999077 China

**Keywords:** aggregation‐induced emission derivative, biomimetic nanoparticles, COVID‐19, cytokine absorption, multimodal therapy, photothermal inactivation

## Abstract

The pandemic of coronavirus disease 2019 (COVID‐19) is continually worsening. Clinical treatment for COVID‐19 remains primarily supportive with no specific medicines or regimens. Here, the development of multifunctional alveolar macrophage (AM)‐like nanoparticles (NPs) with photothermal inactivation capability for COVID‐19 treatment is reported. The NPs, made by wrapping polymeric cores with AM membranes, display the same surface receptors as AMs, including the coronavirus receptor and multiple cytokine receptors. By acting as AM decoys, the NPs block coronavirus from host cell entry and absorb various proinflammatory cytokines, thus achieving combined antiviral and anti‐inflammatory treatment. To enhance the antiviral efficiency, an efficient photothermal material based on aggregation‐induced emission luminogens is doped into the NPs for virus photothermal disruption under near‐infrared (NIR) irradiation. In a surrogate mouse model of COVID‐19 caused by murine coronavirus, treatment with multifunctional AM‐like NPs with NIR irradiation decreases virus burden and cytokine levels, reduces lung damage and inflammation, and confers a significant survival advantage to the infected mice. Crucially, this therapeutic strategy may be clinically applied for the treatment of COVID‐19 at early stage through atomization inhalation of the NPs followed by NIR irradiation of the respiratory tract, thus alleviating infection progression and reducing transmission risk.

## Graphical abstract

A therapeutic strategy for COVID‐19, based on multifunctional alveolar macrophage (AM)‐like nanoparticles (NPs), is successfully developed. The NPs display various AM receptors for coronavirus neutralization and proinflammatory cytokines absorption, thus achieving combined antiviral and anti‐inflammatory treatment. Furthermore, the photothermal feature of the NPs facilitates virus disruption under near‐infrared irradiation. This multimodal therapeutic regimen may be clinically applied for COVID‐19 management.

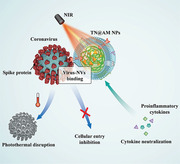

## 1 Introduction

Coronavirus disease 2019 (COVID‐19), caused by severe acute respiratory syndrome coronavirus 2 (SARS‐CoV‐2) infection, has become a severe global public health crisis with high morbidity and mortality, and the pandemic is continually worsening.^[^ ^1^ ^]^ At present, there are still no specific medicines or regimens for the treatment of COVID‐19 in the clinic, and the current paradigm remains primarily supportive.^[^ ^2^ ^]^ Antiviral drugs, such as remdesivir,^[^ ^3^ ^]^ chloroquine,^[^ ^4^ ^]^ and hydroxychloroquine,^[^ ^5^ ^]^ have a potential therapeutic effect on COVID‐19, but their mortality benefit is unproven. The main clinical manifestation of COVID‐19 is severe viral pneumonia, and many critical patients will suffer from acute respiratory distress syndrome (ARDS)^[^ ^6^ ^]^ and multiple organ damage.^[^ ^7^ ^]^ Typically, the violent immune response to viral infection causes a severe inflammatory syndrome (termed “cytokine storm”) in patients, which is closely related to rapid clinical deterioration and may be the primary cause of mortality of COVID‐19.^[^ ^8^ ^]^ Therefore, the development of effective therapeutic strategies with both antiviral and anti‐inflammatory activities are highly desirable.

Cell membrane‐coated nanoparticles (NPs) have recently emerged as a biomimetic nanomedicine platform, offering numerous therapeutic opportunities through cell‐mimicking properties and multifaceted biointerfacing.^[^ ^9^ ^]^ These biomimetic NPs, containing cell membrane protein components, including a variety of pathogen‐related receptors, have been widely applied in the therapeutics of various diseases such as tumors,^[^ ^10^ ^]^ sepsis,^[^ ^11^ ^]^ and virus infection.^[^ ^12^ ^]^ Specifically, NPs coated with CD4+ T cell membranes can neutralize human immunodeficiency virus (HIV) infectivity.^[^ ^12^ ^]^ In addition, NPs coated with macrophage membranes can absorb proinflammatory cytokines for sepsis management.^[^ ^11^ ^]^ Therefore, the biomimetic nanomedicine platform has great potential for neutralizing SARS‐CoV‐2 infectivity, as well as alleviating the “cytokine storm” caused by SARS‐CoV‐2 infection.

Alveolar macrophages (AMs) provide the first line of defense for the host immune system against SARS‐CoV‐2 infection, and the SARS‐CoV‐2 are initially phagocytosed by the AMs when the virions are inhaled into the alveoli.^[^ ^13^ ^]^ The autopsy of COVID‐19 patients shows that large quantities of SARS‐CoV‐2 particles accumulate inside the AMs.^[^ ^14^ ^]^ These results indicate that AMs may be one of the target cells for SARS‐CoV‐2. Additionally, various cytokine receptors, such as IL‐6, TNF‐*α*, or IFN‐*γ* receptors, are expressed on the surface of AM membrane,^[^ ^15^ ^]^ which facilitates the absorption of a variety of cytokines. Therefore, we eventually chose AM membrane as the raw material to prepare biomimetic NPs for COVID‐19 treatment. Since SARS‐CoV‐2 is sensitive to heat,^[^ ^16^ ^]^ we also developed a photothermal strategy for SARS‐CoV‐2 photothermal inactivation to enhance the therapeutic effect of these biomimetic NPs. Biomaterials with photothermal ability hold great potential for the treatment of many diseases, such as tumors,^[^ ^17^ ^]^ infectious diseases,^[^ ^18^ ^]^ and virus infection.^[^ ^19^ ^]^ Here, a highly efficient photothermal material derived from aggregation‐induced emission luminogens (AIEgens), termed 2TPE‐2NDTA,^[^ ^20^ ^]^ was doped into the polymeric cores of biomimetic NPs for virus synergistic photothermal disruption under near‐infrared (NIR) laser irradiation.

In a surrogate mouse model of COVID‐19 caused by murine hepatitis virus A‐59 (MHV‐A59),^[^ ^21^ ^]^ treatment with multifunctional AM‐like NPs (termed “TN@AM NPs”) coupled with NIR irradiation decreased virus burden and cytokine levels, reduced lung damage and inflammation, and conferred a significant survival advantage to the infected mice. For practical application, a new therapeutic regimen, through atomization inhalation of TN@AM NPs followed by NIR irradiation of the respiratory tract, was developed for the clearance of coronavirus residing in the respiratory tract. This regimen was focused on the treatment of coronavirus infection at an early stage, when a large quantity of coronavirus particles are accumulated in the respiratory tract.^[^ ^22^ ^]^ After treatment, the virus burden and cytokine levels in the lungs were significantly decreased, and tissue inflammation and damage were significantly inhibited. Therefore, this therapeutic regimen may be clinically applied for the treatment of COVID‐19 at early stage through the clearance of SARS‐CoV‐2 residing in the respiratory tract, thus alleviating COVID‐19 progression and reducing transmission risk. Furthermore, owing to the abundant receptors expressed on these NPs, this work also opens the door for the management of a broad‐spectrum respiroviral infections, including mutated SARS‐CoV‐2 and emerging viral species infections.

## 2 Results and Discussion

**Scheme 1:**
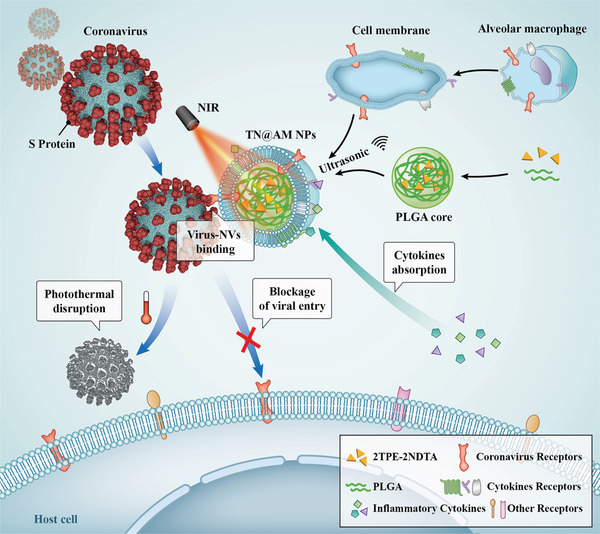
Schematic illustration of multifunctional alveolar macrophage‐like nanoparticles for coronavirus cellular entry blockage, virus photothermal disruption, and inflammatory cytokines absorption.

**Scheme** **1** fully illustrates the principle of multifunctional AM‐like NPs for the management of coronavirus infectious disease (including COVID‐19). After coating AM cell membranes on polymeric cores of poly(lactic‐co‐glycolic acid) (PLGA cores) doped with the highly efficient photothermal molecule 2PTE‐2NDTA^[^ ^20^ ^]^ by sonication, TN@AM NPs are successfully obtained. TN@AM NPs display the same protein receptors required for coronavirus cellular entry and cytokine binding as the source cells. Typically, coronavirus receptors (angiotensin‐converting enzyme 2 (ACE2) for SARS‐CoV‐2^[^ ^23^ ^]^ or CD66a for MHV^[^ ^24^ ^]^) on TN@AM NPs specifically bind to the Spike protein of coronavirus and divert the virus away from their intended host targets, thus inhibiting virus cellular infection. To enhance the antiviral effect, the NP‐virus complex is treated with NIR laser irradiation for coronavirus photothermal inactivation. Meanwhile, the TN@AM NPs are able to absorb a variety of proinflammatory cytokines through various cytokine receptors expressed on the surface of the NPs for the treatment of cytokine storm, which is caused by the violent immune response to coronavirus infection.

### 2.1 Physicochemical and Protein Receptor Characterizations of TN@AM NPs

**Figure 1:**
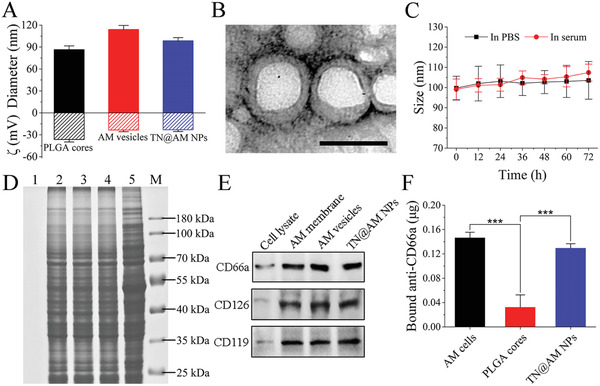
Physicochemical and membrane protein characterizations of TN@AM NPs. A) Hydrodynamic size and surface zeta potential comparison among PLGA cores, AM membrane‐derived vesicles (AM vesicles), and TN@AM NPs, as measured by dynamic light scattering (DLS). Data was presented as mean + standard deviations (SD) (*n* = 3). B) Transmission electron microscopy (TEM) images of TN@AM NPs negatively stained with uranyl acetate. Scale bar: 100 nm. C) Size changes of TN@AM NPs in PBS or fetal bovine serum as determined by DLS over a span of 72 h. Data was presented as mean ± SD (*n* = 3). D) SDS‐PAGE protein analysis of PLGA cores (lane 1), AM membranes (lane 2), AM vesicles (lane 3), TN@AM NPs (lane 4), and AM cell lysate (lane 5). Samples were run at equivalent protein concentrations and stained with Coomassie Blue. Lane M: marker. E) Western blotting analysis for CD66a, CD126, and CD119 receptors in AM cell lysate, AM membranes, AM vesicles, and TN@AM NPs. Samples were run at equal protein concentration. F) Comparison of the fluorescence intensity measured from AM cells (100 µL, ≈2.5 × 10^6^ cells), PLGA cores (100 µL, 1.0 mg mL^−1^), or TN@AM NPs (100 µL, 0.5 mg mL^−1^ protein concentration) stained with FITC‐labeled anti‐CD66a antibodies. Data was presented as mean + SD (*n* = 3). Statistical analysis was conducted using repeated‐measure one‐way analysis of variance (ANOVA) followed by a Tukey post‐hoc test, ***p < 0.001.

To fabricate TN@AM NPs, cytoplasmic membranes of mouse AM cells, namely MH‐S cell line, were derived and purified with combined hypotonic lysis, mechanical membrane disruption, and differential centrifugation. Concurrently, PLGA cores, doped with a highly efficient photothermal material, 2TPE‐2NDTA,^[^ ^20^ ^]^ were synthesized through a modified nanoprecipitation process^[^ ^25^ ^]^ by adding the organic phase of PLGA and 2TPE‐2NDTA to an aqueous phase, followed by organic solvent evaporation. Finally, AM membranes were fused onto PLGA cores by mixing the two components, followed by sonication to fabricate TN@AM NPs. As measured by dynamic light scattering (DLS), the PLGA cores were ≈86 nm in hydrodynamic diameter. Upon fusion of AM membranes with the PLGA cores, the diameter of the final TN@AM NPs increased from 86.4 ± 4.7 to 98.6 ± 4.0 nm and the surface zeta potential increased from −36.4 ± 3.0 to −23.1 ± 2.2 mV (**Figure** **1**A). An increase of ≈12 nm in diameter and ≈13 mV in surface zeta potential is consistent with the addition of a bilayer membrane onto the exterior of the PLGA cores. Membrane coating around the PLGA cores was visualized using transmission electron microscopy (TEM), and the resulting TN@AM NPs were spherical and exhibited a typical core–shell structure (Figure 1B). In contrast, the naked PLGA cores showed no membrane coating, while the AM vesicles, which were fabricated by sonicating the solution of AM membranes alone, had no cores (Figure S1, Supporting Information). Following formulation, TN@AM NPs were suspended in PBS or fetal bovine serum (FBS) and monitored by DLS for 72 h. Under both conditions, TN@AM NPs maintained stable sizes, indicating extended colloidal stability (Figure 1C).

Following the physicochemical characterizations, analysis of the surface protein contents of TN@AM NPs was carried out to confirm successful functionalization of the NPs with AM membrane receptors. First, the protein profiles of the PLGA cores, AM membranes, AM vesicles, TN@AM NPs, and AM cell lysate were analyzed with sodium dodecyl sulfate polyacrylamide gel electrophoresis (SDS‐PAGE). As shown in Figure 1D, the protein profile of TN@AM NPs was modulated when compared to that of the AM cell lysate (including intracellular proteins) but matched closely that of AM membranes and AM vesicles (without intracellular proteins), indicating the preservation of membrane proteins on TN@AM NPs throughout the fabrication process. Cell membrane coating allows TN@AM NPs to inherit receptors related to coronavirus entry and cytokine absorption. For verification, Western blotting analysis showed the presence of the viral receptor CD66a, which mediates MHV cellular entry^[^ ^24^ ^]^ and is similar to the ACE2 receptor that mediates SARS‐CoV‐2 cellular attack, on the TN@AM NPs (Figure 1E). Similarly, cytokine receptors, including CD126 (IL‐6 receptor) and CD119 (IFN‐*γ* receptor), were also present in AM cell lysate, AM membranes, AM vesicles, and TN@AM NPs (Figure 1E), indicating potential for proinflammatory cytokine absorption and anti‐cytokine storm therapy caused by coronavirus infection. These results also showed that the AM vesicle and TN@AM NP preparations significantly facilitated membrane protein retention and enrichment, further confirming the translocation of AM membranes and associated membrane proteins onto nanoparticle surfaces (Figure 1E). The asymmetric repulsion between the PLGA cores and the extracellular membrane versus the intracellular membrane during the cell membrane coating process determines the right‐side‐out membrane orientation,^[^ ^26^ ^]^ which is essential for viral neutralization and cytokine adsorption. To examine this orientation in TN@AM NPs, flow cytometry was first performed on the AM cells through surface staining by fluorescence‐labeled anti‐CD66a antibodies, and the outer surface of AM cells showed high expression of CD66a (Figure S2, Supporting Information). Then, we stained TN@AM NPs and AM cells containing equal amounts of membrane content using fluorescently labeled antibodies against the CD66a molecule. PLGA cores served as a blank control. After removing free antibodies, the NP sample showed fluorescence intensity comparable with that of the AM cell sample, while the naked PLGA cores showed low antibody binding (Figure 1F). As inside‐out membrane coating would likely block antibody staining and reduce fluorescence intensity, comparable fluorescence intensity between TN@AM NPs and AM cells suggests that TN@AM NPs adopted primarily a right‐side‐out membrane orientation.

### 2.2 Photothermal Performance of TN@AM NPs

**Figure 2:**
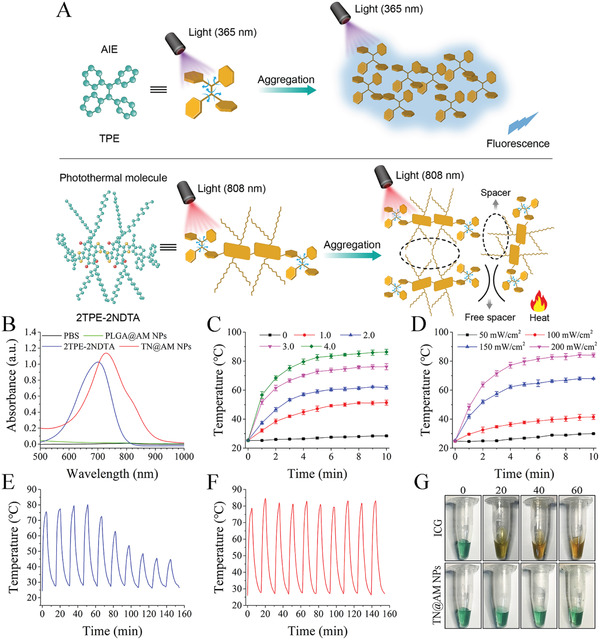
Photothermal performance of TN@AM NPs. A) A sketch map of working mechanisms of TPE (conventional AIEgen) and 2TPE‐2NDTA (AIEgen‐derived photothermal material). B) UV–vis absorption spectra comparison among PBS, PLGA@AM NP, 2TPE‐2NDTA (in THF), and TN@AM NP solutions. C) Photothermal performance of TN@AM NPs at different concentrations (protein concentration, mg mL^−1^) upon exposure to 808 nm laser irradiation (200 mW cm^−2^) for different times. D) Photothermal performance of TN@AM NPs (4.0 mg mL^−1^ protein concentration) upon exposure to different 808 nm laser power densities for different times. E,F) Photothermal cycles of ICG (0.16 mg mL^−1^) and TN@AM NP (4.0 mg mL^−1^ protein concentration) solutions upon exposure to 808 nm laser irradiation (200 mW cm^−2^). G) Photographs of ICG and TN@AM NP solutions after persistent 808 nm laser irradiation (300 mW cm^−2^) for different times (min). In (C, D), data were presented as mean ± SD (*n* = 3).

In this study, a highly efficient organic photothermal molecule with donor–acceptor structures, termed 2TPE‐2NDTA,^[^ ^20^ ^]^ was doped into the PLGA cores of TN@AM NPs for coronavirus photothermal disruption under NIR laser irradiation (Scheme 1). The molecular design of 2TPE‐2NDTA (Figure S3, Supporting Information) was based on a conventional AIEgen, namely tetraphenylethylene (TPE),^[^ ^27^ ^]^ as it undergoes active excited‐state intramolecular motion in the free state. AIEgens are a special type of molecule that are non‐emissive in the dissolved state but are induced to emit bright fluorescence by aggregation due to the restriction of intramolecular motions.^[^ ^28^ ^]^ The acceptors of 2TPE‐2NDTA are naphthalene diimide‐fused 2‐(1,3‐dithiol‐2‐ylidene)acetonitriles (2NDTAs) with long alkyl chains,^[^ ^29^ ^]^ which enable the intermolecular spatial isolation of the molecules in the aggregate state to produce some necessary space to promote free intramolecular motion (**Figure** **2**A).^[^ ^30^ ^]^ This structure may explain the phenomenon that 2TPE‐2NDTA exhibited highly efficient photothermal conversion without fluorescence emission by taking advantage of active intramolecular motion in the aggregate state within NPs, while TPE displays bright fluorescence emission without photothermal properties by restriction of active excited‐state intramolecular motion in the aggregate state (Figure 2A).

The chemical structure and synthetic route of 2TPE‐2NDTA are presented in Figures S3,S4, Supporting Information. The structure of 2TPE‐2NDTA was confirmed by NMR spectroscopy, with satisfactory results (Figures S5,S6, Supporting Information). UV–vis absorption spectra analysis showed that the TN@AM NP solution presented a characteristic absorption peak centered at ≈720 nm, similar to the 2TPE‐2NDTA solution. In contrast, the PLGA@AM NP (without 2TPE‐2NDTA inside) solution and PBS showed no absorption peaks, indicating the successful encapsulation of 2TPE‐2NDTA inside TN@AM NPs (Figure 2B). The NIR absorption feature of 2TPE‐2NDTA, results from the large *π*‐conjugation and strong electron‐withdrawing ability of the acceptors,^[^ ^31^ ^]^ which holds great potential for clinical applications due to the deep tissue penetration of NIR light. To determine the highest loading capability of TN@AM NPs, different weight of 2TPE‐2NDTA were added in the NPs preparation process. After preparation, the amount of the loaded 2TPE‐2NDTA in the TN@AM NPs was calculated utilizing the concentration‐absorbance standard curve of 2TPE‐2NDTA (Figure S7, Supporting Information). As shown in Table S1 (Supporting Information), when 0.80 mg of 2TPE‐2NDTA was added for the NPs preparation, the amount of encapsulated 2TPE‐2NDTA reach the highest level, while adding extra 2TPE‐2NDTA did not increase the loading amount anymore. Therefore, we eventually chose 0.80 mg of 2TPE‐2NDTA for the preparation of TN@AM NPs. Additionally, NPs derived from red blood cell (RBC) membranes (termed “RBC NPs”) were prepared as a control. The encapsulation efficiencies of 2TPE‐2NDTA in TN@AM NPs and RBC NPs were 92.5% ± 4.9% and 93.8% ± 5.8%, respectively. The drug loading ratios of 2TPE‐2NDTA in TN@AM NPs and RBC NPs were 4.16% ± 0.22% or 4.24% ± 0.28%, respectively.

The photothermal performance of TN@AM NPs (Figure 2C,D) and RBC NPs (Figure S8, Supporting Information) at different concentrations or under different irradiation power densities of an 808 nm laser were examined. Notably, the maximum plateau temperature of the TN@AM NP solution (4.0 mg mL^−1^ protein concentration) or the RBC NP solution (4.0 mg mL^−1^ protein concentration) reached ≈82 °C or ≈84 °C, respectively, within 6 min of 808 nm laser irradiation (200 mW cm^−2^). In addition, the photothermal conversion efficiency of TN@AM NPs was comparable to that of indocyanine green (ICG) (Figure S9, Supporting Information), which has been approved by the Food and Drug Administration for clinical use.^[^ ^32^ ^]^ More importantly, the solution temperature reached ≈50 °C at a very low concentration of TN@AM NPs (1.0 mg mL^−1^ protein concentration) (Figure 2C). These results demonstrate that TN@AM NPs can rapidly and efficiently convert NIR energy into heat, and their photothermal conversion efficiency holds great potential for photothermal disruption of SARS‐CoV‐2, which is sensitive to heat.^[^ ^16^ ^]^

In addition, the photothermal stability of TN@AM NPs was also investigated. As shown in Figure 2E,F, the temperature elevation of the ICG solution gradually decreased during several cycles of 808 nm irradiation (200 mW cm^−2^), while the temperature elevation of the TN@AM NP solution remained almost unchanged. After constant irradiation with a high density of 808 nm laser (300 mW cm^−2^) for 60 min, the extrinsic feature of the TN@AM NP solution remained unchanged, while the extrinsic color of the ICG solution was altered (Figure 2G). Furthermore, the UV–vis absorbance results showed that ICG was degraded under persistent NIR irradiation (300 mW cm^−2^), while 2TPE‐2NDTA was undegraded (Figure S10, Supporting Information). These results indicate that TN@AM NPs are stable enough to serve as an excellent photothermal agent.

### 2.3 TN@AM NPs Inhibit MHV‐A59 Entry and Infection of Host Cells

**Figure 3:**
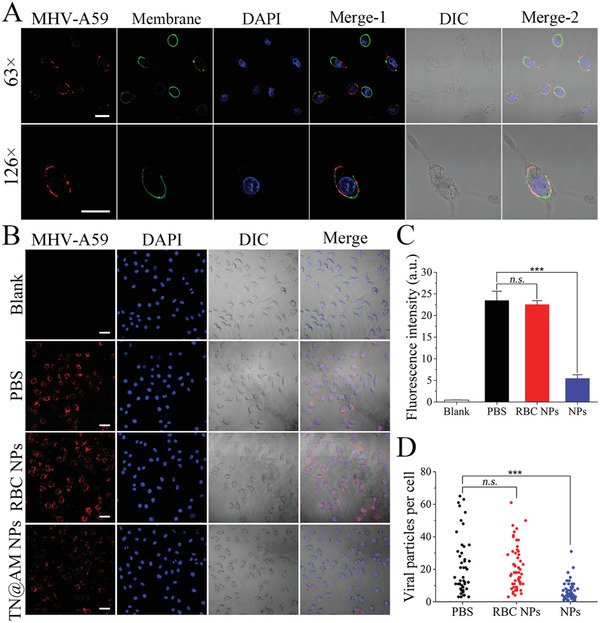
TN@AM NPs inhibit MHV‐A59 cellular infection. A) Confocal laser scanning microscopy (CLSM) images for MHV‐A59 binding to the surface of AM membrane after coincubation for 10 min. The MHV‐A59 particles were labeled with quantum dots (red); The AM membrane was stained with DiO dye (green); The AM cell nuclei were stained with DAPI (blue). Scale bars: 20 µm. B) MHV‐A59 cellular entry inhibition analysis of PBS, RBC NPs (4.0 mg mL^−1^ protein concentration) and TN@AM NPs (4.0 mg mL^−1^ protein concentration). L929 cells as the target cells. L929 cells without MHV‐A59 or treatment was set as a blank control. The MHV‐A59 particles were labeled with quantum dots (red); The L929 cell nuclei were stained with DAPI (blue). Scale bars: 100 µm. C) Fluorescence intensity analysis for the quantum dot‐labeled MHV‐A59 particles among different treatments in (B). Data were presented as mean + SD (*n* = 4). D) Counts of MHV‐A59 particle localization in single L929 cells among different treatments in (B). Statistical analysis was conducted using one‐way ANOVA followed by a Tukey post‐hoc test, ***p < 0.001, *n.s*.: not significant.

We have proven that the specific receptor CD66a (like the ACE2 receptor for SARS‐CoV‐2 cellular infection^[^ ^23^ ^]^), which mediates MHV cellular entry,^[^ ^24^ ^]^ is present on the surface of TN@AM NPs (Figure 1E,F). We next investigated the binding capability of AM membranes to MHV‐A59 particles and the inhibition ability of TN@AM NPs against MHV‐A59 cellular invasion. As shown in **Figure** **3**A and Figure S11 (Supporting Information), quantum dot‐labeled MHV‐A59 particles (red) specifically bound to the surface of the AM membrane stained with DiO dye (green) after coincubation for only 10 min at room temperature, indicating the high binding capability of MHV‐A59 to AM membranes. To evaluate whether TN@AM NP treatment could prevent MHV‐A59 from infecting susceptible host cells, a mixture of MHV‐A59 (1 × 10^5^ plaque‐forming units, PFU) and TN@AM NPs (4.0 mg mL^−1^ protein concentration) was first incubated at 37 °C for 1 h for MHV‐NP binding and then added to L929 cells for another 1 h incubation for MHV‐A59 absorption onto the host cell membranes. After removing the supernatant followed by washing with PBS for three times, the L929 cells were incubated for another 6 h for MHV‐A59 cellular entry. L929 cells without MHV‐A59 infection and PBS treatment served as blank and positive controls, respectively. L929 cells without infection or treatment served as a blank control. PBS treatment served as a mock control.RBC NP treatment served as a comparison control, which showed no CD66a expression compared to TN@AM NPs (Figure S12, Supporting Information). As shown in Figure 3B and Figure S13 (Supporting Information), TN@AM NPs significantly inhibited MHV‐A59 cellular invasion, while RBC NPs showed no inhibition abilities, compared to the PBS group. In the presence of TN@AM NPs, the average fluorescence signals of quantum dots labeled MHV‐A59 inside L929 cells significantly decreased, while the average fluorescence intensity after RBC NP treatment showed no difference compared to the PBS group, which indicated that MHV‐A59 cellular infection was significantly inhibited by TN@AM NPs (Figure 3C). The number of MHV‐A59 particles per single cell was also analyzed. There were ≈20 virions per cell after treatment with RBC NPs, which showed no difference to the PBS group, while the number of virions decreased to ≈5 viruses per cell after the TN@AM NP treatment (Figure 3D). These results indicate that TN@AM NPs significantly inhibit MHV‐A59 entry and infection of host cells, which may result from the blockage of the viral Spike protein by the CD66a receptor present on TN@AM NPs.

### 2.4 The Antivirus and Anticytokine Ability of TN@AM NPs In Vitro

**Figure 4:**
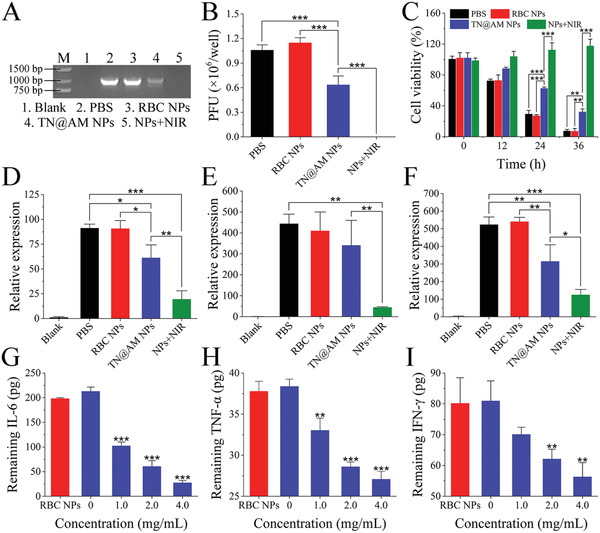
The antivirus and anticytokine ability of TN@AM NPs in vitro. A) Detection of the MHV‐A59 burden in L929 cell lysate through standard RT‐PCR amplification of the MHV‐A59 genome sequence after different treatments followed by virus replication for 24 h. B) Detection of the MHV‐A59 burden in L929 cell supernatants through a standard plaque assay after different treatments followed by virus replication for 24 h. C) Cell viability analysis of L929 cells after different treatments followed by virus replication for 24 h (CCK‐8 used as an indicator). Detection of proinflammatory cytokine mRNA expression in RAW264.7 cell lysates through a standard RT‐qPCR assay after different treatments followed by cellular inflammatory stimulation by MHV‐A59 for 24 h: The cytokines included D) IL‐6, E) IL‐1*β*, and F) G‐CSF. G–I) Removal of proinflammatory cytokines, including IL‐6, TNF‐*α*, and IFN‐*γ*, with indicated concentration of TN@AM NPs. RBC NPs (4.0 mg mL^−1^) was set as a control. All data were presented as mean + SD (*n* = 3). Statistical analysis was conducted using one‐way ANOVA followed by a Tukey post‐hoc test, *p < 0.05, **p < 0.01, ***p < 0.001.

We next investigated the inhibitory effects of TN@AM NPs on viral propagation in vitro. The mixture of MHV‐A59 (1 × 10^5^ PFU) and TN@AM NPs (4.0 mg mL^−1^ protein concentration) was first incubated at 37 °C for 1 h for MHV‐NP binding, followed with (“NPs+NIR” group) or without (“AM@TN NPs” group) subsequent 808 nm laser (200 mW cm^−2^) irradiation for 5 min. Then, the mixture was added to L929 cells for 1 h incubation for MHV‐A59 cellular infection. After removing the supernatant followed by washing with PBS for three times, the L929 cells were incubated for another 24 h for MHV‐A59 replication. L929 cells without MHV‐A59 infection or treatment served as the blank control (“Blank” group). PBS treatment served as the mock control (“PBS” group). RBC NP treatment served as a comparison control (“RBC NPs” group). After 24 h of different treatments, the virus burden in the L929 cell lysates was detected by standard RT‐PCR amplification of the MHV‐A59 genome sequence, while the virus burden in the L929 cell supernatants was detected by a standard plaque assay. As shown in **Figure** **4**A,B, TN@AM NP treatment moderately decreased the MHV‐A59 burden compared to the PBS group, while “NPs+NIR” treatment nearly eradicated the MHV‐A59 load due to virus photothermal disruption. However, the RBC NP treatment showed no antiviral abilities. Additionally, treatment of TN@AM NPs coupled with NIR irradiation completely eliminated L929 cell death caused by MHV‐A59 infection, while TN@AM NPs alone delayed the cell death process after MHV‐A59 infection compared to that in “PBS” and “RBC NPs” groups (Figure 4C). The morphological changes of L929 cells also showed that MHV‐A59 infection caused cell disruption, while the extrinsic features of L929 cells remained unchanged after “NPs+NIR” treatment (Figure S14, Supporting Information).

Furthermore, we proceeded to test the anticytokine ability of TN@AM NPs. After the different treatments described above, the mixture of MHV‐A59 and NPs was added to mouse macrophage RAW264.7 cells for another 24 h of cellular inflammatory stimulation. Macrophages were chosen as the target cells because this type of immunocyte will produce a large quantity of cytokines and is the main driver of cytokine storms caused by SARS‐CoV‐2 infection.^[^ ^33^ ^]^ After 24 h, the relative expression of multiple proinflammatory cytokines of different treatments from the RAW264.7 cell lysates was detected by standard RT‐qPCR. As shown in Figure 4D–F, TN@AM NP treatment moderately decreased the expression of cytokines compared to that in “PBS” and “RBC NPs” groups, while TN@AM NPs with NIR treatment significantly decreased the expression level of proinflammatory cytokines, including IL‐6, IL‐1*β*, and G‐CSF. It has been reported that macrophage‐like NPs act as decoys to absorb proinflammatory cytokines and can efficiently inhibit the systemic inflammatory response caused by sepsis.^[^ ^11^ ^]^ To test the cytokine adsorption potential of TN@AM NPs, recombinant IL‐6, TNF‐*α* and IFN‐*γ* were incubated with different concentrations of TN@AM NPs. After ultracentrifugation to discard the NPs, the supernatant was analyzed by ELISA to determine the concentration of unconjugated cytokines. RBC NPs (4.0 mg mL^−1^ protein concentration) served as a comparison group. As shown in Figure 4G–I, TN@AM NPs displayed tremendous cytokine adsorption power for IL‐6, TNF‐*α*, and IFN‐*γ* compared to that of the RBC NPs, in a concentration‐dependent manner. These results indicate that TN@AM NPs hold tremendous potential for the treatment of the SARS‐CoV‐2 infection‐induced cytokine storm, which is closely related to rapid clinical deterioration and may be the main driver of mortality for COVID‐19 patients.^[^ ^34^ ^]^

### 2.5 Toxicity and Biocompatibility Evaluation of TN@AM NPs

**Figure 5:**
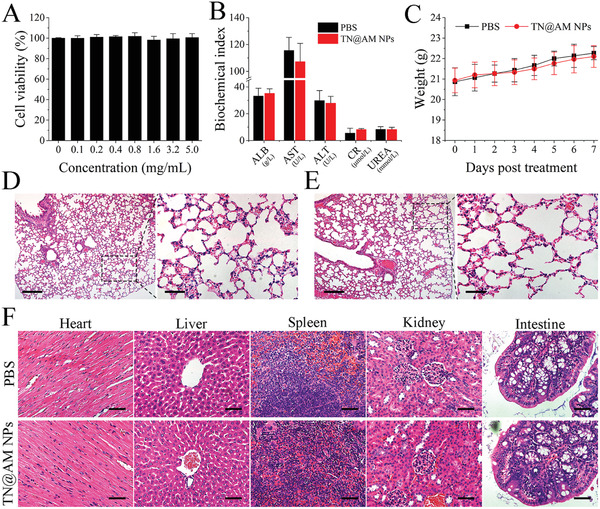
Toxicity evaluation of TN@AM NPs in vitro and in vivo. A) Cell viability analysis after incubation of different concentrations of TN@AM NPs with normal mouse alveolar epithelial MLE‐12 cells for 24 h (CCK‐8 used as an indicator). Data were presented as mean + SD (*n* = 6). B) Liver and kidney function biochemistry index comparison in healthy mice after intranasal administration of TN@AM NPs or PBS for 7 days. Data were presented as mean + SD (*n* = 4). C) Body weight changes of healthy mice after intranasal administration of TN@AM NPs or PBS for different days. Data were presented as mean ± SD (*n* = 6). Hematoxylin‐eosin (HE) staining analysis of mice lung tissues after intranasal administration of D) PBS or E) TN@AM NPs for 7 days. Scale bars (left panels): 200 µm. Scale bars (right panels): 50 µm. F) HE staining analysis of various important mice organs after intranasal administration of TN@AM NPs or PBS for 7 days. Scale bars: 50 µm. In (B–F), healthy mice were intranasally administered with 7.5 mg kg^−1^ TN@AM NPs in a suspension of 30 µL or 30 µL PBS.

To facilitate the application of TN@AM NPs in vivo, their toxicity and biocompatibility must be analyzed. As shown in **Figure** **5**A, upon incubation of normal mouse alveolar epithelial MLE‐12 cells with TN@AM NPs for 24 h, the cell viability did not decrease regardless of the concentration of NPs used (up to 5.0 mg mL^−1^ protein concentration), suggesting little cytotoxicity of TN@AM NPs to normal lung epithelial cells. After intranasal administration of TN@AM NPs (30 µL, 7.5 mg kg^−1^ based on protein weight) to healthy mice, biochemical indexes reflecting hepatic function including albumin (ALB), aspartate aminotransferase (AST), and alanine transaminase (ALT) levels, as well as renal‐related indexes including creatinine (Cr) and blood urea nitrogen (UREA) levels, were tested. No physiologically significant difference was observed between the TN@AM NPs and PBS group at 7 days post administration (Figure 5B), indicating that TN@AM NPs caused no apparent dysfunction in the liver and kidney. In addition, the body weight changes of mice in the NP and PBS groups were monitored for 7 days, and no noticeable body weight loss was observed in either group (Figure 5C). Furthermore, hematoxylin‐eosin (HE) staining of lungs (Figure 5D,E) and other vital organs (Figure 5F) revealed no pathological change after NP administration for 7 days. These results demonstrate that TN@AM NPs are safe biomaterials with no toxicity for in vivo applications.

### 2.6 Therapeutic Efficiency Evaluation of TN@AM NPs in a Surrogate Mouse Model of COVID‐19

The animal experiment is essential to evaluate the therapeutic effect of TN@AM NPs. However, wild‐type mice are not susceptible to SARS‐CoV‐2 infection^[^ ^35^ ^]^ because mouse ACE2 receptor cannot bind to the Spike protein of SARS‐CoV‐2.^[^ ^36^ ^]^ Thus, there is an urgent need to develop a surrogate mouse model of COVID‐19 for SARS‐CoV‐2 research. The murine coronavirus MHV‐A59, fits well within the betacoronavirus group,^[^ ^37^ ^]^ which includes SARS‐CoV‐2,^[^ ^38^ ^]^ was therefore chosen as a potential surrogate virus for SARS‐CoV‐2 research. Intranasal infection with MHV‐A59 has been reported to serve as a surrogate mouse model of severe pneumonia caused by severe acute respiratory syndrome coronavirus (SARS‐CoV) or Middle East respiratory syndrome coronavirus (MERS‐CoV) infections.^[^ ^21^ ^]^ Severe lung histopathological alterations are evident in this surrogate mouse model, including edema, congestion, diffuse alveolar damage, pneumocyte desquamation, inflammatory leukocyte infiltration, and alveolar wall thickening.^[^ ^21^ ^]^ Typical elevations in the levels of various proinflammatory cytokines, such as IL‐6, TNF‐*α*, IFN‐*γ*, IL‐1*β*, and IP‐10,^[^ ^21^ ^]^ were found in this animal model, which closely mimics the cytokine storm caused by coronavirus infection. Herein, we speculated that this model could be regarded as a surrogate mouse model of COVID‐19.

**Figure 6:**
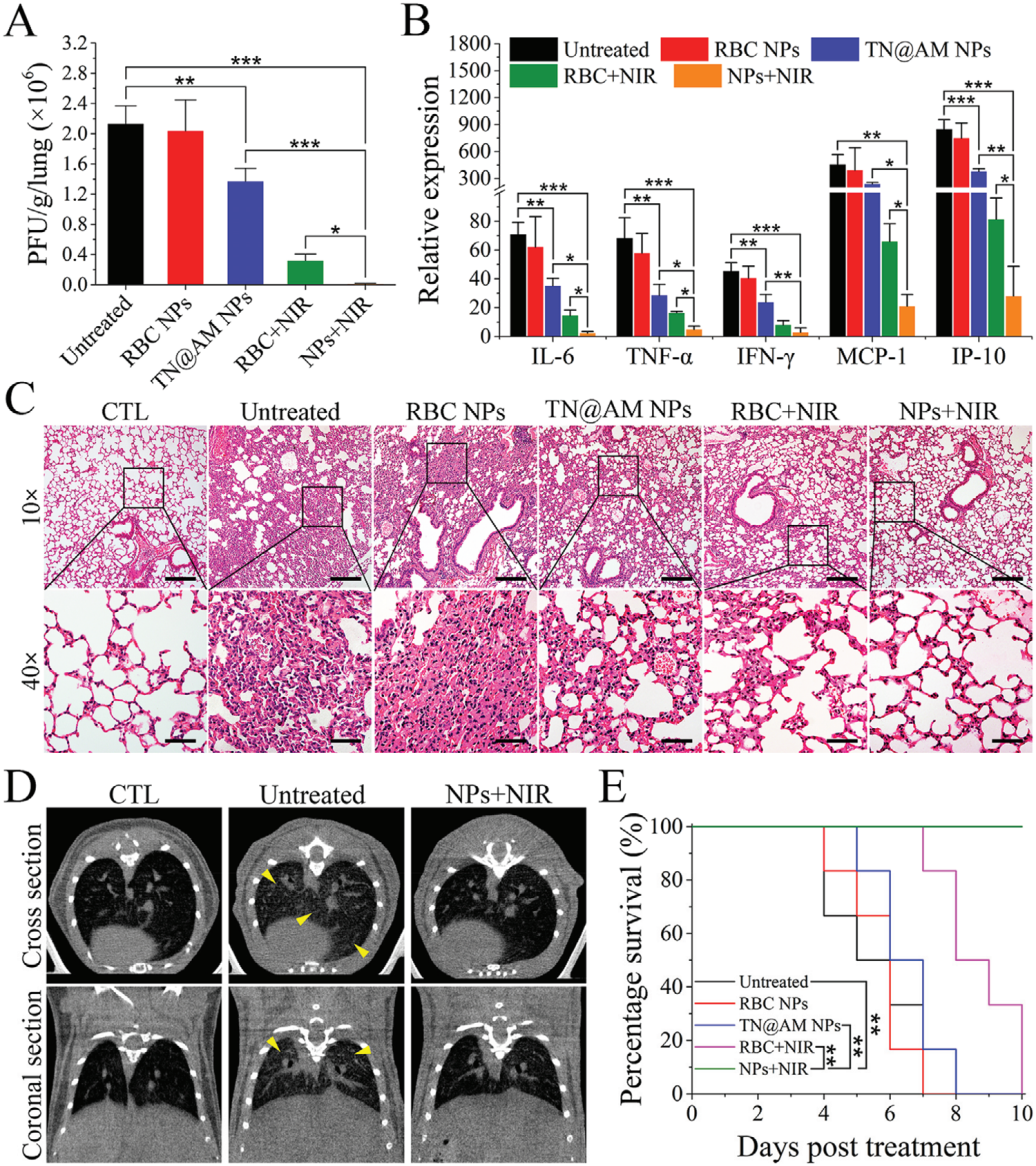
Therapeutic effect analysis of TN@AM NPs in vivo. A) Detection of MHV‐A59 burden in the lung tissues through a standard plaque assay at 5 days post different treatments. B) Detection of the mRNA expression of various proinflammatory cytokines in the lung tissues through a standard RT‐qPCR assay at 5 days post different treatments. C) HE staining analysis of lung tissues at 5 days post different treatments. Scale bars (10×): 200 µm. Scale bars (40×): 50 µm. D) Computed tomography (CT) comparison of mouse lungs at 5 days post different treatments. Yellow arrowheads indicate the ground‐glass opacities. E) Survival analysis of mice receiving different treatments (*n* = 6 for each group). In (A, B), all data were presented as mean + SD (*n* = 3), statistical analysis was conducted using one‐way ANOVA followed by a Tukey post‐hoc test. In (E), statistical analysis was conducted using Gehan–Breslow–Wilcoxon test; *p < 0.05, **p < 0.01, ***p < 0.001.

We thus evaluated the therapeutic effect of TN@AM NPs in this surrogate mouse model of COVID‐19. In the study, 5 × 10^5^ PFU of MHV‐A59 was first incubated with TN@AM NPs or RBC NPs (4.0 mg mL^−1^ protein concentration) at 37 °C for 1 h, followed with (“NPs+NIR” and “RBC+NIR” groups) or without (“TN@AM NPs” and “RBC NPs” groups) subsequent NIR irradiation (200 mW cm^−2^) for 5 min. Then, the mixtures of MHV‐A59 and NPs were intranasally inoculated into 6‐week‐old BALB/c mice for lung infection. Mice without infection or treatment served as the blank control (“CTL” group). Mice infected with MHV‐A59 alone served as the mock control (“Untreated” group). After 5 days of different treatments, the virus burden, the mRNA expression of various proinflammatory cytokines, the inflammatory infiltration, and the tissue damage in the mice lungs were detected. As shown in **Figure** **6**A, TN@AM NP treatment decreased the lung virus burden to some degree compared to the untreated group, while RBC NP treatment showed limited antiviral ability. In addition, treatment with TN@AM NPs coupled with NIR irradiation (“NPs+NIR” group) significantly decreased the MHV‐A59 burden in the lungs compared to the TN@AM NP group, and almost eradicated the infection. However, compared to the “NPs+NIR” treatment, the “RBC+NIR” group showed less antiviral efficacy. This was based on the fact that TN@AM NPs decreased the distance between the MHV‐A59 and the heating cores (2TPE‐2DNTA doped PLGA cores) through Spike protein‐CD66a binding, which did not happen between the virions and the heating cores of RBC NPs.

The anticytokine ability analysis demonstrated that the TN@AM NP treatment inhibited proinflammatory cytokines to some degree compared to the untreated group. In contrast, the RBC NP treatment showed no anti‐cytokine efficiency. Furthermore, the “NPs+NIR” treatment significantly decreased the expression of various proinflammatory cytokines (IL‐6, TNF‐*α*, IFN‐*ϒ*, MCP‐1, IP‐10) activated by MHV‐A59 infection compared to the “TN@AM NPs” and “RBC+NIR” groups (Figure 6B).This anticytokine ability results from the photothermal disruption of MHV‐A59 upon NIR irradiation and cytokine absorption by the AM membrane coating on the surface of TN@AM NPs, indicating that the “NPs+NIR” strategy holds tremendous clinical potential for the treatment of cytokine storm caused by SARS‐CoV‐2 infection. Furthermore, lung histopathological alterations were analyzed among different treatments (Figure 6C). Typically, severe lung damage was observed after MHV‐A59 infection (“Untreated” group), including edema, diffuse alveolar damage, inflammatory leukocyte infiltration, and alveolar wall thickening. Treatment with TN@AM NPs reduced lung damage to some degree, while treatment with RBC NPs showed no remission ability. Additionally, treatment with TN@AM NPs coupled with NIR irradiation significantly reduced lung damage compared to the “TN@AM NPs” and “RBC+NIR” groups, and the HE staining results were nearly as normal as those of healthy mice (“CTL” group). More importantly, “NPs+NIR” treatment significantly improved the pulmonary computed tomography (CT) results compared to those in the untreated group (Figure 6D). The pulmonary CT result of untreated mice presented severe ground‐glass opacities, indicating severe inflammatory infiltration and exudation, which was the major CT changes observed in critical COVID‐19 patients.^[^ ^39^ ^]^ Furthermore, survival and weight change analysis showed that the “NPs+NIR” treatment completely prevented mice weight loss and death. However, treatment with RBC NPs, TN@AM NPs, or RBC NPs coupled with NIR irradiation showed limited survival and weight loss advantages (Figure 6E; Figure S15, Supporting Information).

Pretreating MHV‐A59 with TN@AM NPs coupled with NIR irradiation before intranasal inoculation of the NP‐virus mixtures into mice is not a practical application. Therefore, we next investigated the therapeutic effect of TN@AM NPs on mice already infected with MHV‐A59 . We have proved that the antiviral and anti‐inflammatory abilities of TN@AM NPs alone were not sufficient, while TN@AM NPs coupled with NIR irradiation showed significant therapeutic effects (Figures 4 and 6). Thus, NIR irradiation was essential for virus and inflammation clearance, and the “NPs+NIR” strategy held tremendous potential for the treatment of COVID‐19. However, COVID‐19 patients at the late stage of SARS‐CoV‐2 infection usually suffer from diffused infectious lesions rather than local ones in the lungs,^[^ ^40^ ^]^ and irradiating the whole lung with NIR laser cannot be achieved. Additionally, there may be potential risks after NIR irradiation of the chest, which contains many vital organs such as the heart and lungs. Therefore, the “NPs+NIR” strategy cannot be applied for the direct antiviral and anti‐inflammatory therapy in the lungs.

**Figure 7:**
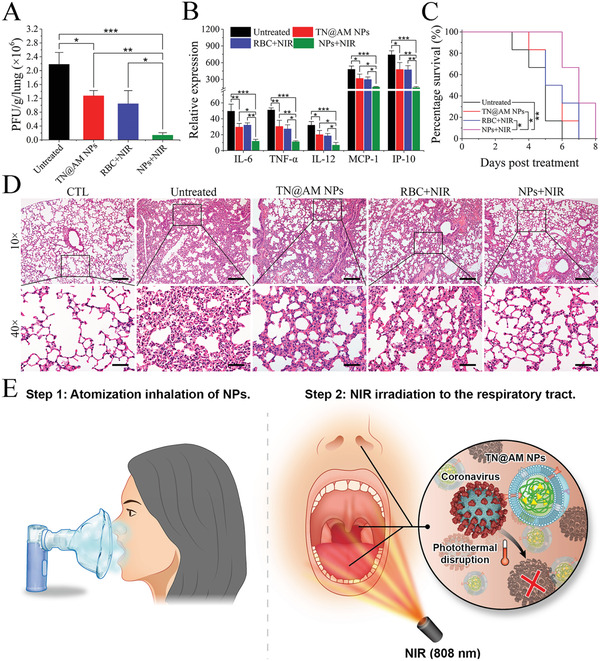
Therapeutic effect of TN@AM NPs in vivo after atomization inhalation of the NPs followed by NIR irradiation of the respiratory tract. A) Detection of MHV‐A59 burden in the lung tissues through a standard plaque assay at 5 days post different treatments. B) Detection of the mRNA expression of various proinflammatory cytokines in the lung tissues through a standard RT‐qPCR assay at 5 days post different treatments. C) Survival analysis of mice receiving different treatments (*n* = 6 for each group). D) HE staining analysis of lung tissues at 5 days post different treatments. Scale bars (10×): 200 µm. Scale bars (40×): 50 µm. E) Schematic diagram of anti‐SARS‐CoV‐2 therapeutic regimen through atomization inhalation of the TN@AM NPs followed by NIR irradiation of the respiratory tract. In (A) and (B), all data were presented as mean + SD (*n* = 3), statistical analysis was conducted using one‐way ANOVA followed by a Tukey post‐hoc test. In (C), statistical analysis was conducted using Gehan–Breslow–Wilcoxon test; *p < 0.05, **p < 0.01, ***p < 0.001.

SARS‐CoV‐2 is transmitted mainly through respiratory droplets. After inhalation by healthy individuals, a large quantity of SARS‐CoV‐2 will initially reside in the respiratory tract (the early stage of infection).^[^ ^22^ ^]^ Subsequently, SARS‐CoV‐2 will replicate in the respiratory tract, the infection will spread to the lungs, and the transmission abilities of these infected patients will increase.^[^ ^13^ ^]^ Considering that the TN@AM NPs developed in this study are mainly targeted at extracellular SARS‐CoV‐2 rather than infected host cells, this TN@AM NPs based strategy is also suitable for the treatment of COVID‐19 at early infection stage. Therefore, we designed the following therapeutic regimen based on TN@AM NPs. The mice were first infected with MHV‐A59 (5 × 10^5^ PFU) by atomization inhalation for 30 min, modeling the early stage of MHV‐A59 infection in the respiratory tract. Then TN@AM NPs (7.5 mg kg^−1^ protein weight) were administered by atomization inhalation. After 1 h of NP‐virus binding, the mouse respiratory tract (through the nasal cavity and oral cavity) was irradiated with 808 nm (200 mW cm^−2^) laser for 15 min for photothermal disruption of MHV‐A59. Mice without MHV‐A59 infection served as a blank control (“CTL” group). Mice infected with MHV‐A59 alone served as the mock control (“Untreated” group). Mice treated with TN@AM NPs alone (“TN@AM NPs” group) or RBC NPs coupled with NIR irradiation (“RBC+NIR” group) served as comparison groups. Finally, the virus burden and cytokine levels in the lungs (at 5 days post different treatments), the mouse survival rates and the lung tissue damages among different groups were analyzed. As shown in **Figure** **7**A, treatment of TN@AM NPs coupled with NIR irradiation (“NPs+NIR” treatment) significantly decreased the lung virus burden compared to the “Untreated” “TN@AM NPs” and “RBC+NIR” groups. Meanwhile, the anticytokine ability analysis demonstrated that “NPs+NIR” treatment significantly reduced the levels of various proinflammation cytokines, including IL‐6, TNF‐*α*, IL‐12, MCP‐1, and IP‐10, compared to those in the“Untreated,” “TN@AM NPs,” and “RBC+NIR” groups (Figure 7B). Furthermore, survival analysis showed that, compared to the “Untreated,” “TN@AM NPs,” or “RBC+NIR” treatments, “NPs+NIR” treatment significantly improved the survival time of infected mice (Figure 7C). However, treatment with TN@AM NPs alone or RBC NPs coupled with NIR irradiation showed limited survival advantages compared to the “Untreated” group. More importantly, lung histopathological alterations were analyzed among different treatments. Typically, “NPs+NIR” treatment significantly reduced lung damage and inflammatory leukocyte infiltration compared to those in the “Untreated”, “TN@AM NPs” or “RBC+NIR” groups (Figure 7D). These results indicated that the clearance of virus colonized in the respiratory tract through the “NPs+NIR” treatment could alleviate virus replication, inflammatory activation, and infection progression in the lungs.

It has been reported that therapeutic regimens through laser irradiation of the respiratory tract have been widely applied for the treatment of respiratory bacterial infection,^[^ ^41^ ^]^ tumor,^[^ ^42^ ^]^ and virus infection.^[^ ^43^ ^]^ Thus, the above “NPs+NIR” strategy may also be extended to clinical COVID‐19 therapeutics, especially for early‐stage SARS‐CoV‐2 infection. Therefore, we conceive of a novel treatment regimen utilizing “NPs+NIR” administration for COVID‐19 therapy and prevention. As presented in Figure 7E, patients at the early stage of SARS‐CoV‐2 infection are first administered with TN@AM NPs into the respiratory tract through atomization inhalation, and then the TN@AM NPs will bind to SARS‐CoV‐2 residing in the respiratory tract through specific receptor‐ligand binding. After virus‐NP binding, an NIR laser is used to irradiate the respiratory tract directly through the nasal and oral cavities or through fiber bronchoscopes to realize SARS‐CoV‐2 photothermal disruption and clearance. This treatment regimen, namely "atomization inhalation of TN@AM NPs followed by NIR irradiation of the respiratory tract" regimen, holds tremendous potential for the therapy of patients at the early stage of SARS‐CoV‐2 infection, especially those with positive nucleic acid tests of throat swabs but with no clinical symptoms, thus reducing the spread of SARS‐CoV‐2 to the lungs and other tissues, alleviating COVID‐19 progression, reducing disease severity, and decreasing patients mortalities. More importantly, this "atomization inhalation of TN@AM NPs followed by NIR irradiation of the respiratory tract" treatment regimen can significantly restrict viral transmission and holds potential for COVID‐19 prevention.

## 3 Conclusions

Multifunctional AM‐like NPs (termed TN@AM NPs) were successfully fabricated for combined antiviral and anti‐inflammatory treatments in a surrogate disease of COVID‐19 caused by mouse coronavirus infection. Specifically, the TN@AM NPs display the same surface receptors, required for coronavirus cellular entry (such as CD66a for MHV cellular entry) and cytokine binding (such as IL‐6 receptor and IFN‐*γ* receptor), as the source cells. By acting as decoys, these NPs selectively bind with MHV‐A59 and effectively inhibit MHV‐A59 host cell invasion. Owing to the highly efficient photothermal material (named 2TPE‐2NDTA) doped in the PLGA cores of the NPs, the TN@AM NPs successfully achieved virus photothermal disruption under NIR irradiation. Furthermore, the TN@AM NPs displayed efficient absorption of multiple proinflammatory cytokines, thus hold tremendous potential for the treatment of the cytokine storm caused by coronavirus infection. In a surrogate mouse model of COVID‐19, treatment with TN@AM NPs coupled with NIR irradiation significantly inhibited viral replication, decreased proinflammatory cytokine levels, reduced lung damage, and ultimately conferred a survival advantage to infected mice. Crucially, this biomimetic antivirus strategy can be clinically applied for the clearance of SARS‐CoV‐2 residing in the respiratory tract through atomization inhalation of the NPs followed by NIR irradiation of the respiratory tract, thus alleviating COVID‐19 progression, reducing disease severity, decreasing patient mortality, and reducing the transmission risk. Moreover, this work also opens the door to utilizing cell membrane‐coating technology for the management of broad‐spectrum respirovirus infections, including mutated SARS‐CoV‐2 and emerging viral species infections.

## Conflict of Interest

The authors declare no conflict of interest.

## Supporting information

Supporting Information

## Data Availability

Research data are not shared.
