# Supplementary material for: Antiviral and Anti‐Inflammatory Treatment with Multifunctional Alveolar Macrophage‐Like Nanoparticles in a Surrogate Mouse Model of COVID‐19
Source: Adv Sci (Weinh). 2021 May 12;8(13):2003556. doi: 10.1002/advs.202003556 (PMC8209923; doi:10.1002/advs.202003556)
Supplement: Supplementary file 1 — Supporting Information [file ADVS-8-0-s001.pdf]

© 2021 Wiley-VCH GmbH

ADVANCED SCIENCE
Open Access

Supporting Information

for *Adv. Sci.,* DOI: 10.1002/advs.202003556

Antiviral and Anti-Inflammatory Treatment with Multifunctional Alveolar Macrophage-like Nanoparticles in a Surrogate Mouse Model of COVID-19

Bin Li‡, Wei Wang‡, Weifeng Song‡, Zheng Zhao‡, Qingqin Tan, Zhaoyan Zhao, Lantian Tang, Tianchuan Zhu, Jialing Yin, Jun Bai, Xin Dong, Siyi Tan, Qunying Hu, Ben Zhong Tang*, and Xi Huang**

Supporting Information

Antiviral and Anti-Inflammatory Treatment with Multifunctional Alveolar Macrophage-like Nanoparticles in a Surrogate Mouse Model of COVID-19

Bin Li[‡]*, Wei Wang*[‡]*, Weifeng Song*[‡]*, Zheng Zhao*[‡]*, Qingqin Tan, Zhaoyan Zhao, Lantian Tang, Tianchuan Zhu, Jialing Yin, Jun Bai, Xin Dong, Siyi Tan, Qunying Hu*[*]*, Ben Zhong Tang*[*]*, and Xi Huang*[*]

B. Li, W. Wang, W. F. Song, Q. Q. Tan, Z. Y. Zhao, L. T. Tang, T. C. Zhu, J. L. Yin, J. Bai, X. Dong, S. Y. Tan, Prof. X. Huang
Center for Infection and Immunity
Guangdong Provincial Key Laboratory of Biomedical Imaging
The Fifth Affiliated Hospital of Sun Yat-sen University
Zhuhai 519000, Guangdong, China
E-mail: huangxi6@mail.sysu.edu.cn

B. Li, Q. Q. Tan, Z. Y. Zhao, L. T. Tang, T. C. Zhu, J. L. Yin, J. Bai, X. Dong, S. Y. Tan, Prof. X. Huang
Southern Marine Science and Engineering Guangdong Laboratory
Zhuhai 519000, Guangdong, China

Dr. Z. Zhao, Prof. B. Z. Tang
Department of Chemistry
The Hong Kong University of Science and Technology
Clear Water Bay, Kowloon 999077, Hong Kong, China
E-mail: tangbenz@ust.hk

W. Wang, Prof. Q. Y. Hu
Xizang Minzu University
Xianyang 712082, Shanxi, P. R. China.
E-mail: huqunying2020@163.com

‡These authors contributed equally to this work.

Experimental Section

Alveolar Macrophage Membrane Derivation.

MH-S cells (murine alveolar macrophage cell line) was purchased from the American Type Culture Collection (ATCC) and cultured in the RPMI 1640 medium (Gibco) supplemented with 10% fetal bovine serum (Hyclone) and 1% penicillin-streptomycin (Gibco) in suspension flasks. Plasma membrane was collected according to a previously published method. Specifically, cells were grown in T-175 culture flasks to high density and the cells were collected by centrifugation at 700 × g for 5 min. The cells were washed with 1×PBS three times (500 × g for 10 min each) and the cell pellet was dispersed in homogenization buffer containing 75 mM sucrose, 20 mM Tris·HCl (pH=7.5, MediaTech), 2 mM $MgCl_2$ (Sigma), 10 mM KCl (Sigma), and one tablet of protease/phosphatase inhibitors (Pierce, Thermo Fisher Scientific). The suspension was loaded into a dounce homogenizer and the cells were disrupted with 15-25 passes. Following the disruption, the suspension was spun down at 800 × g for 5 min to remove large debris. The supernatant was collected and centrifuged again at 10000 × g for 25 min, after which the pellet was discarded, and the supernatant was centrifuged at 150000 × g for 50 min. After the centrifugation, the supernatant was discarded, and the plasma membrane was collected as an off-white pellet. The membrane pellet was then washed once with 1×10^{-3} M ethylenediaminetetraacetic acid (EDTA; Sigma) in H_2O, and resuspended with gentle sonication for subsequent experiments. Membrane protein content was quantified with a Pierce BCA assay (Life Technology).

Preparation and Characterization of TN@AM NPs.

TN@AM NPs were formulated in two steps. In the first step, PLGA cores containing 2TPE-2NDTA were prepared using 0.67 dL g^{-1} carboxyl-terminated 50:50 PLGA (LACTEL absorbable polymers) through a nanoprecipitation method. The PLGA polymer and 2TPE-2NDTA were first dissolved in acetone at a concentration of 10 mg mL^{-1} and 0.5 mg mL^{-1}, respectively. Then 1 mL of the solution was added rapidly to 4 mL of water. The nanoparticle solution was then stirred in open air for 3 h

to remove the organic solvent. In the second step, the collected alveolar macrophage membranes were mixed with PLGA cores at a membrane protein-to-polymer weight ratio of 1:1. The mixture was sonicated with a Fisher Scientific FS30D bath sonicator at a frequency of 42 kHz and a power of 100 W for 3 min. TN@AM NPs were purified from free vesicles, membrane fragments, and unbound proteins by centrifugation at 16,000 × g for 30 min. Nanoparticles were measured for size and size distribution with DLS (ZEN 3600 Zetasizer, Malvern). All measurements were done in triplicate at room temperature. Serum and PBS stabilities were examined by mixing 1 mg mL^{-1} protein concentration of TN@AM NPs with 1× FBS and 2× PBS, respectively, at a 1:1 volume ratio. Membrane coating was confirmed with transmission electron microscopy (TEM). Briefly, 3 μL of nanoparticle suspension (1 mg mL^{-1}) was deposited onto a glow-discharged carbon-coated copper grid. After 5 min, the grid was rinsed with 10 drops of distilled water, followed by staining with a drop of 1 wt% uranyl acetate. The grid was subsequently dried and visualized using a JEM-1400 PLUS 120 kV transmission electron microscope.

Characterization of Membrane Proteins.

Protein profiles of PLGA cores, cell lysate, cell membranes, AM vesicles and TN@AM NPs were characterized with sodium dodecyl sulfate polyacrylamide gel electrophoresis (SDS-PAGE). Specifically, samples were prepared at a protein concentration of 2.0 mg mL^{-1} in lithium dodecyl sulfate (LDS) loading buffer (Invitrogen), heated at 100°C for 15 min, and then loaded into Bolt 10% Bis-Tris Plus Gels (Invitrogen). Electrophoresis was carried out in the MOPS buffer system (Invitrogen) with an XCell SureLock Electrophoresis System (Invitrogen) per manufacturer's instruction. Following the electrophoresis, the gel was immersed in SimplyBlue buffer (Invitrogen) for 1 h to stain the proteins. For western blot analysis, AM cell lysate, AM cell membranes, AM vesicles, and TN@AM NPs were mixed with lithium dodecyl sulfate (LDS) loading buffer to the same total protein concentration of 2 mg mL^{-1}. Electrophoresis was carried out with NuPAGE Novex 6-12% Bis-Tris 15-well minigels in Mops running buffer with an XCell SureLock

Electrophoresis System (Invitrogen). Then the protein was transferred onto Nitrocellulose membranes (Whatman) in NuPAGE transfer buffer (Invitrogen) at 70 V for 2 h. The membranes were blocked for 1 h and then probed with rabbit anti-mouse CD66a (Biolegend), rabbit anti-mouse CD126 (Abcam), and rabbit anti-mouse CD119 (Abcam), respectively. Corresponding IgG-horseradish peroxidase (HRP) conjugates were used for the secondary staining (Biolegend). Films were developed with the ECL western blotting substrate (Pierce) on a Mini-Medical/90 Developer (ImageWorks). To stain the surface proteins for membrane orientation, TN@AM NPs (100 μL, 0.5 mg mL^{-1} protein concentration), PLGA cores (100 μL, 0.5 mg mL^{-1}) or MH-S cells (100 μL, ≈2.5 $\times 10^6$ cells) were blocked in 1% BSA for 30 min, followed by incubation with 0.2 μg fluorescein isothiocyanate (FITC)-labeled anti-CD66a antibody (Abcam) for 30 min. To remove unbound antibodies, MH-S cell samples were spun at 3000 × g for 5 min, whereas TN@AM NPs and PLGA cores samples were centrifuged in Nanosep tubes with a molecular weight cutoff of 300 kDa and a speed of 6000 × g for 2 min. The fluorescence intensity of the unbound antibody was measured and used to calculate the amount of antibodies that bound to the TN@AM NPs, PLGA cores and MH-S cells.

NIR Laser-Induced Heat Conversion.

TN@AM NPs solutions of different concentrations (0, 0.02, 0.04, 0.08, 0.16 mg mL^{-1} based on 2TPE-2NDTA) or upon different 808 nm laser power (0.5, 1.0, 1.5, 2.0 W cm^{-2}, BWT Beijing Ltd, Beijing, China) were prepared to estimate the photothermal conversion performance and photothermal stability. ICG solution (0.16 mg mL^{-1}) was used as control. During irradiation, the FLIR A35 IR thermal imaging camera was employed to monitor the temperature variation.

Preparation of RBC NPs and PLGA@AM NPs.

RBC NPs were prepared according to the protocols for TN@AM NPs preparation. RBC membranes collected from female BALB/c mouse RBCs through hypotonic lysis were coated onto preformed 2TPE-2NDTA doped PLGA cores by sonication.

PLGA@AM NPs were prepared according to the protocols for TN@AM NPs preparing.

Cytokines Binding Studies.

TN@AM NPs samples (0, 1.0, 2.0, 4.0 mg mL^{-1} protein concentration) or RBC NPs (4.0 mg mL^{-1} protein concentration) were mixed with IL-6 (2,000 pg mL^{-1}), TNF-α (400 pg mL^{-1}), or IFN-γ (800 pg mL^{-1}) in PBS containing 10% FBS were incubated at 37°C for 2 h. Following the incubation, the samples were centrifuged at 20,000 × g for 30 min to pellet the nanoparticles. Cytokine concentrations in the supernatant were quantified by using ELISA (BioLegend). All experiments were performed in triplicate.

Viral Loads by Plaque Assay and RT-PCR.

To quantify the infectious virus particles in the lungs, portions of the lungs removed at necropsy were weighed and homogenized in DMEM with 10% FBS, and rapidly frozen and thawed for three times. Cell debris were removed by centrifugation, and the virus titers (PFU per g tissue) in the supernatants were determined by plaque assay on L929 cells. MHV-A59 genome sequence expression in the host cells or the lung was analyzed by RT-PCR. Briefly, total RNA was extracted from 2×10^5 infected L929 cells or 100 mg mice lung tissue with TRIzol reagent (Invitrogen, CA, USA), and 2 μg RNA, pretreated with 1 U of RQ1 RNase-free DNase (Fisher Scientific) to remove DNA contamination at 37 ºC for 30 min, were used for reverse transcription with oligo-dT primer (Promega). PCR primers were derived from the MHV-A59 genome sequence (NCBI NC_001846, nt 5040-6119): forward 5'-CGG AAT TCG GGT TGA TGT CTT GTG TAC TG-3' and reverse 5'-CCG CTC GAG TTA CAA TTT AAA GTT GGT ATAGAC-3'. PCR reactions were then performed using the above primers to detect the MHV-A59 genome sequence. PCR products were resolved by electrophoresis in 1.5% agarose gels and visualized a VersaDoc imaging system (Bio-Rad).

RT-qPCR detection of cytokines expression levels.

Total RNA extraction by Trizol (Invitrogen, CA, USA) and quantitative real-time PCR reactions in a MyiQ cycler (Bio-Rad, CA, USA) using SYBR Green I (Molecular Probes, OR, USA) were performed. The primer pairs for mouse IL-6, IL-1β, TNF-α, IFN-γ, MCP-1, IP-10, G-CSF, β-actin were IL-6 forward: 5'-CACAGA GGATACCACATCCCAACA-3'; IL-6 reverse: 5'-TCCACGATTTCCCAGAGAAC A-3'; IL-1β forward: 5'-CAACCAACAAGTGATATTCTCCATG-3'; IL-1β reverse: 5'-GATCCACACTCTCCAGCTGCA-3'; TNF-α forward: 5'-GGTGCCTATGTCTC AGCCTCTT-3'; TNF-α reverse: 5'-CGATCACCCCGAAGTT CAGTA-3'; IFN-γ forward: 5'-GCTCTGAGACAATGAACGCTAC-3'; IFN-γ reverse: 5'-TTCTTCCA CATCTATGCCACTT-3'; MCP-1 forward: 5'-TGGGTCCAGACATACATT-3'; MCP-1 reverse: 5'-ACGGGTCAACTTCACATT-3'; IP-10 forward: 5'-GCTGGGTCTGAG TGGGA-3'; IP-10 reverse: 5'-ACGTGGGCAGGATAGGC-3'; G-CSF forward: 5'-GTGTTGCTGGGCCACTCT-3'; G-CSF reverse: 5'-CGCTGGAAGGCAGAAGTG-3'; β-actin forward: 5'- GATTACTGCTCTGGCTCCTAGC-3'; β-actin reverse: 5'-GACTCATCGTACTCCTGCTTGC-3'. The mRNA levels of indicated genes were normalized to that of β-actin mRNA.

Establishment of a Surrogate Mouse Model of COVID-19 by MHV-A59 Infection.

All animal experiments were performed in compliance with the guidelines established by the Fifth Affiliated Hospital of Sun Yat-sen University. To establish a surrogate mouse model of COVID-19, 6-week-old BALB/c female mice were anesthetized intraperitoneally with pentobarbital sodium combined with chloral hydrate (Sigma), and then inoculated intranasally with 30 μL of MHV-A59 virus at 5×10^5 plaque-forming unit (PFU), and control mice were inoculated intranasally with 30 μL of PBS. The mice were monitored daily for health conditions.

Therapeutic effect analysis of TN@AM NPs *in vivo*.

MHV-A59 at 5×10^5 PFU was first incubated with TN@AM NPs or RBC NPs (2.0 mg mL^{-1} protein concentration) at 37°C for 1 h. Then the mixture of MHV-A59 and NPs was treated with ("NPs+NIR" and "RBC+NIR" groups) or without ("TN@AM

NPs" and "RBC NPs" groups) NIR irradiation (200 mW cm^{-2}) for 5 min. Afterwards, the mixture was intranasally inoculated into 6-week-old BALB/c mice for lung infection for several days. Mice without infection and treatment served as the blank control ("CTL" group). Mice infected with MHV-A59 alone served as the mock control ("Untreated" group). At 5 days post different treatments, the virus burden and mRNA expression of various proinflammatory cytokines were measured by a standard plaque assay and a standard RT-qPCR assay, respectively. Meanwhile, HE staining analysis of lung tissues and computed tomography (CT) analysis of mice were performed. In addition, the survival analysis of mice after different treatments were performed.

Therapeutic effect analysis of TN@AM NPs *in vivo* after atomization inhalation of the NPs followed by NIR irradiation of the respiratory tract.

Six-week-old BALB/c mice were first infected with 5×10^5 PFU of MHV-A59 by intranasal inoculation for 30 min. Then TN@AM NPs (7.5 mg kg^{-1} protein weight) were administered by atomization inhalation. After 1 h, the mouse respiratory tract (through the nasal cavity and oral cavity) was irradiated with 808 nm (200 mW cm^{-2}) laser for 10 min. Mice without MHV-A59 infection served as a blank control ("CTL" group). Mice infected with MHV-A59 alone served as the mock control ("Untreated" group). Mice treated with TN@AM NPs alone ("TN@AM NPs" group) or RBC NPs coupled with NIR irradiation ("RBC+NIR" group) served as comparison groups. At 5 days post different treatments, the virus burden and mRNA expression of various proinflammatory cytokines were measured by a standard plaque assay and a standard RT-qPCR assay, respectively. Meanwhile, HE staining analysis of lung tissues were performed. In addition, the survival analysis of mice after different treatments were performed.

Statistical Analysis.

The mRNA expression data of proinflammatory cytokines from each were normalized as the ratios to the blank controls. Quantitative data were expressed as mean + SD or mean ± SD. For the antiviral and anti-inflammatory analysis *in vitro*

and *in vivo*, sample size (*n*) = 3 for each group. For survival analysis, $n = 6$ for each group. Statistical comparisons among different groups were determined by one-way ANOVA followed by a post-hoc Tukey's HSD test, and *p* values between each group were adjusted by Bonferroni correction. Statistical analysis of survival rates among different groups were determined by Gehan-Breslow-Wilcoxon test. For all tests, $p < 0.05$ was considered statistically significant; *, **, and *** indicate $p < 0.05$, $p < 0.01$ and $p < 0.001$, respectively. All statistical calculations were performed using GraphPad Prism 5.0 Software, including assumptions of tests used (GraphPad Software Inc., CA, USA).

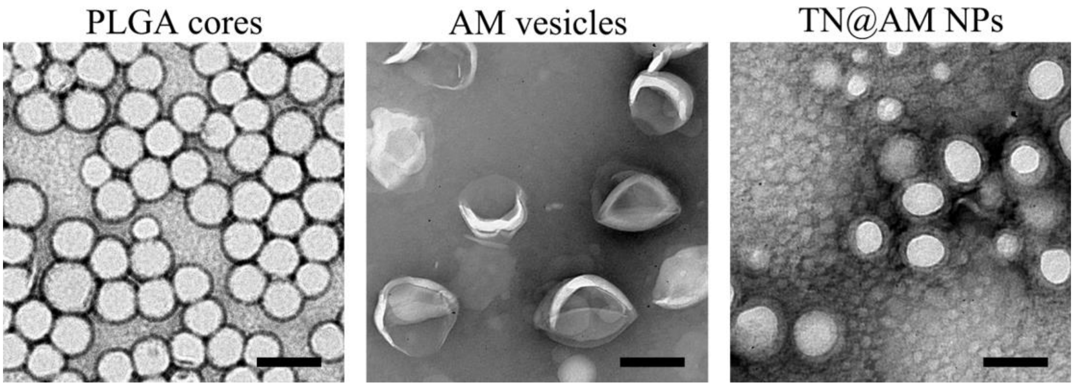

Figure S1. Transmission electron microscopy (TEM) images of PLGA cores, AM vesicles, and TN@AM NPs. Samples were negatively stained with uranyl acetate. Scale bars: 100 nm.

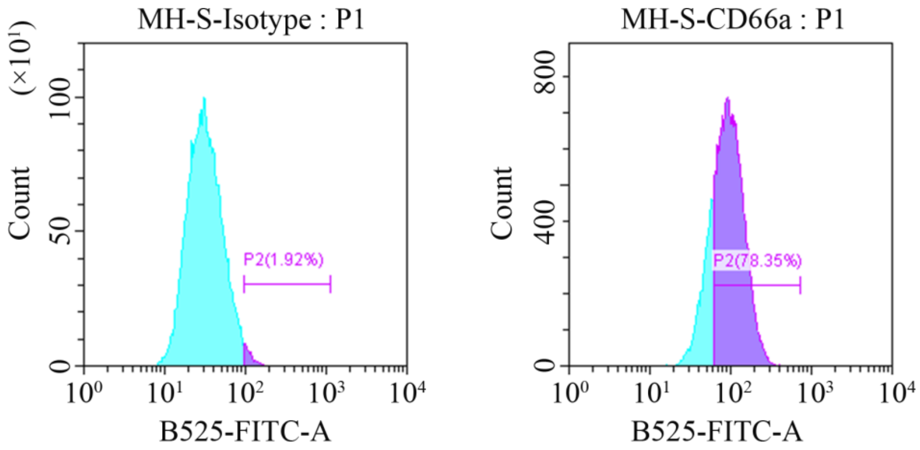

Figure S2. The expression of CD66a on MH-S cells was detected by flow cytometry. MH-S cells were stained with FITC-labeled IgG antibodies (Isotype) or FITC-labeled CD66a antibodies, respectively.

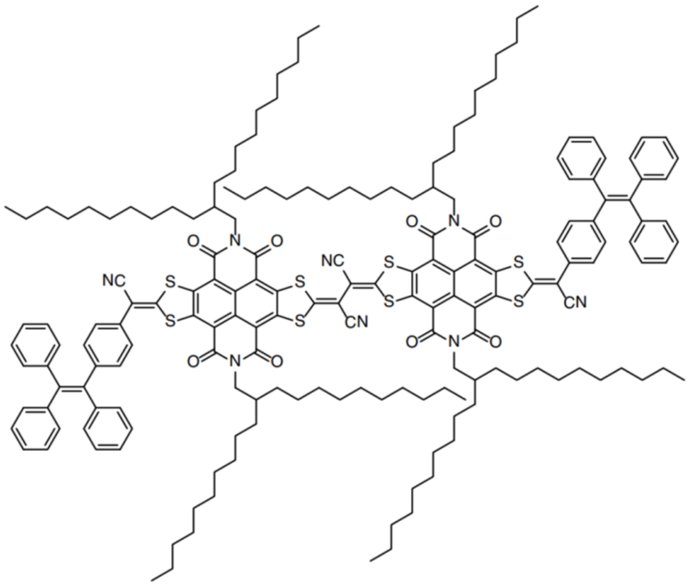

Figure S3. The chemical structure of 2TPE-2NDTA.

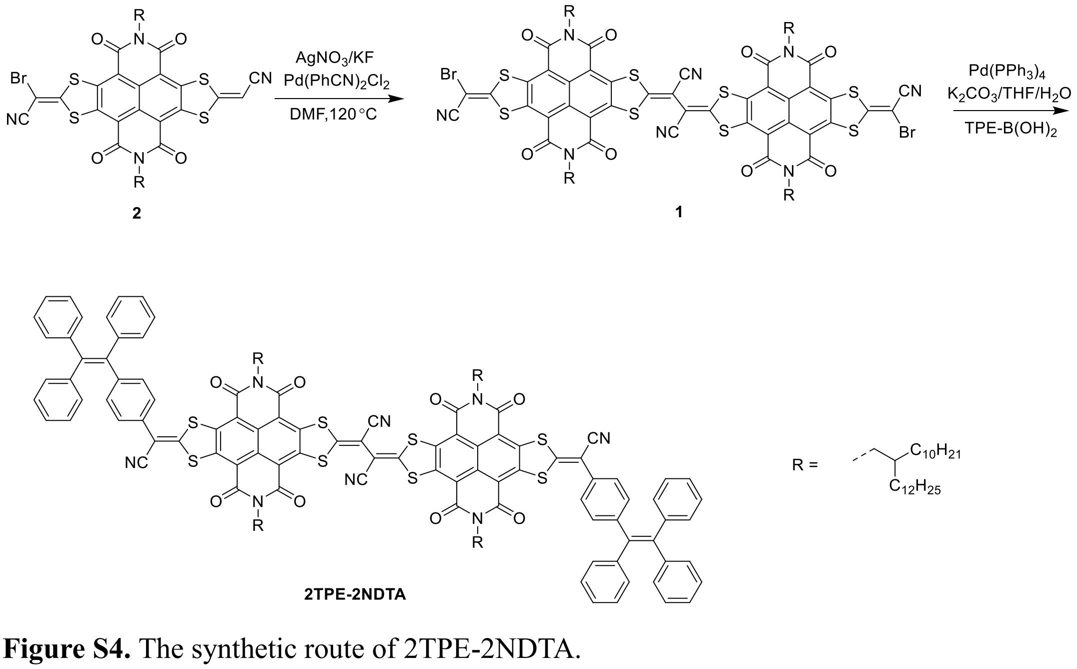

Figure S4. The synthetic route of 2TPE-2NDTA.

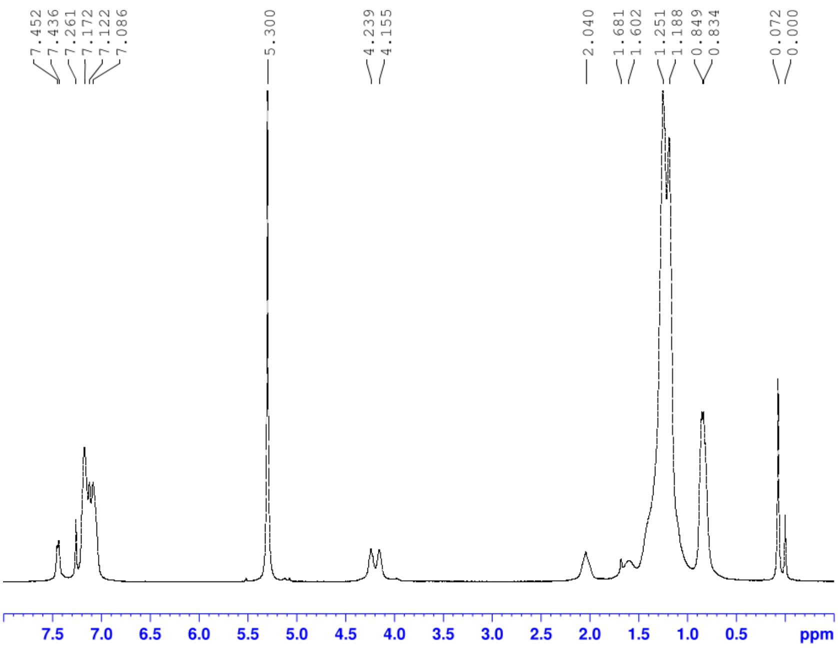

Figure S5. ^{1}H NMR spectrum of 2TPE-2NDTA using the deuterated solvent as the lock and tetramethylsilane (TMS; $\delta = 0$) as internal reference.

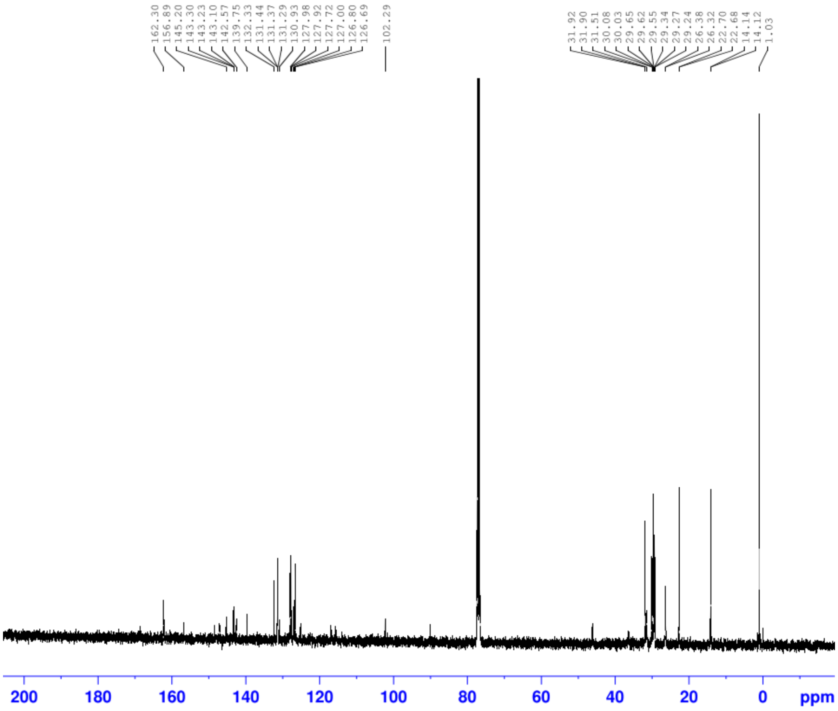

Figure S6. ^{13}C NMR spectrum of 2TPE-2NDTA using the deuterated solvent as the lock and tetramethylsilane (TMS; $\delta = 0$) as internal reference.

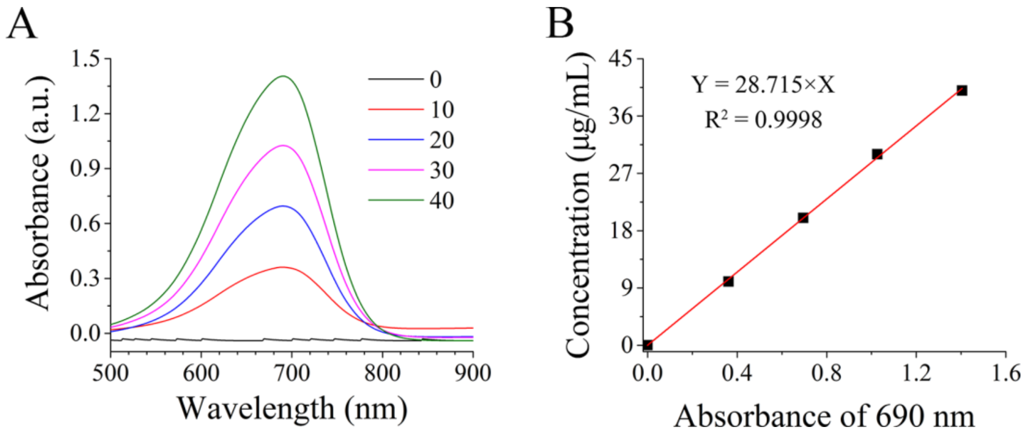

Figure S7. (A) UV-vis absorption spectra of different concentrations of 2TPE-2NDTA (μg mL^{-1}) in THF. (B) The concentration-absorbance standard curve of 2TPE-2NDTA in THF.

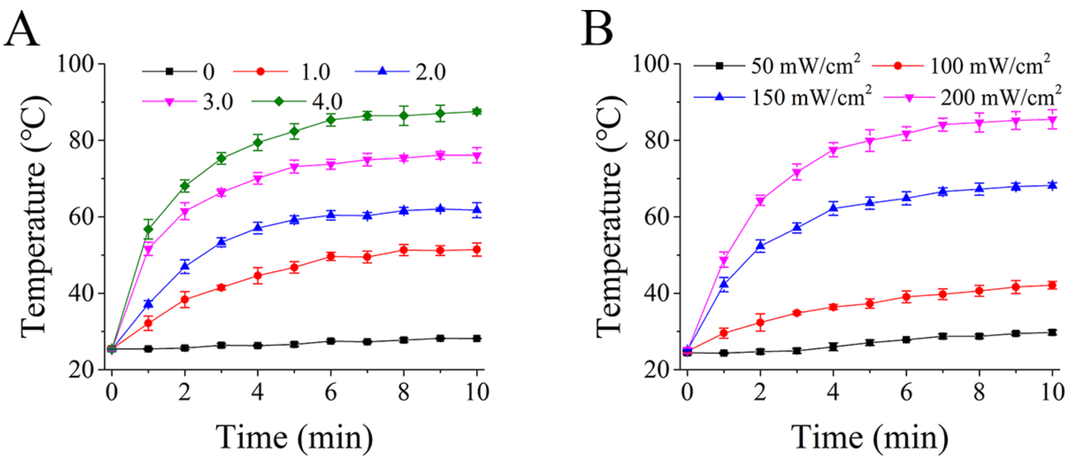

Figure S8. (A) Photothermal performance of RBC NPs at different concentrations (protein concentration, mg mL-1) upon exposure to 808 nm laser irradiation (200 mW cm^{-2}) for different times. (B) Photothermal performance of RBC NPs (4.0 mg mL^{-1} protein concentration) upon exposure to different 808 nm laser power densities for different times.

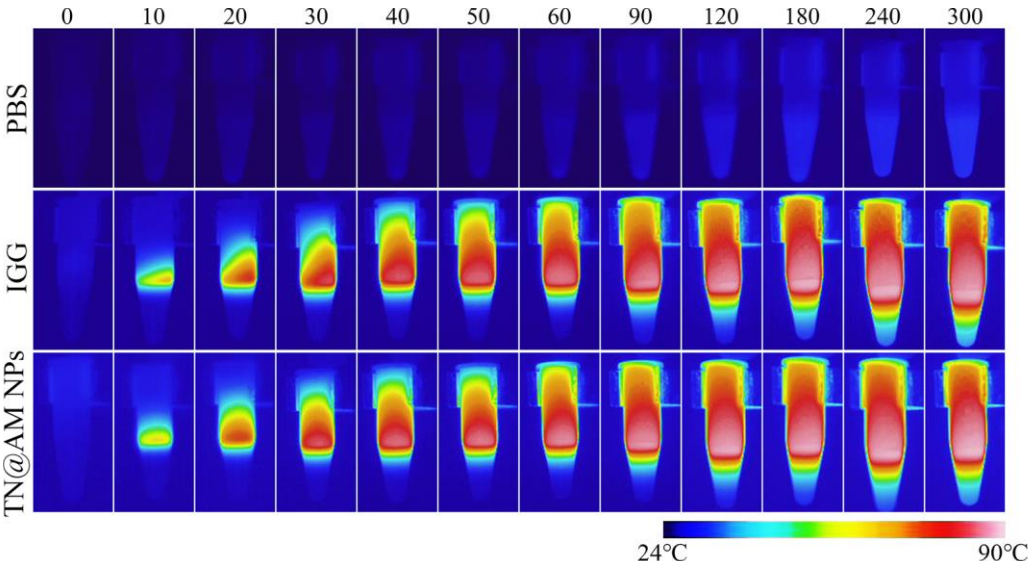

Figure S9. IR thermal images of PBS, indocyanine green (ICG) solution (0.16 mg mL^{-1}) and TN@AM NPs solution (0.16 mg mL^{-1} based on 2TPE-2NDTA) upon exposure to 808 nm (2.0 W cm^{-2}) laser irradiation for different times (s).

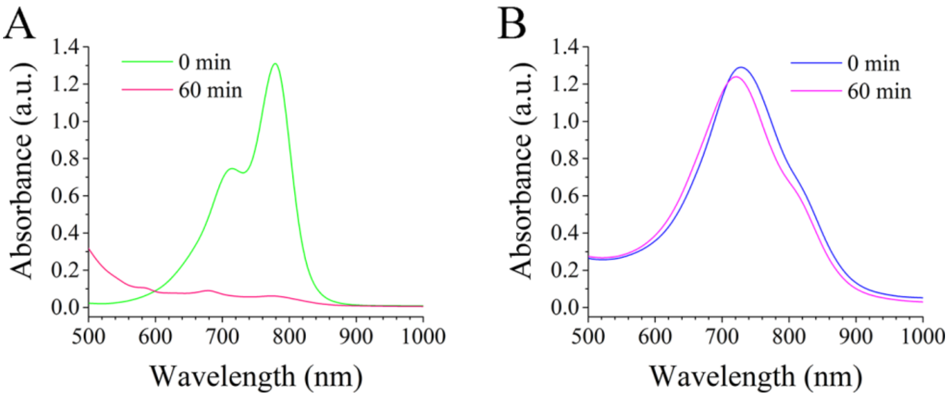

Figure S10. UV-vis absorbance spectra of ICG solution (0.16 mg mL^{-1}) (A) and TN@AM NPs solution (4.0 mg mL^{-1} protein concentration) (B) before or after persistent 808 nm (300 mW cm^{-2}) laser irradiation for 60 min.

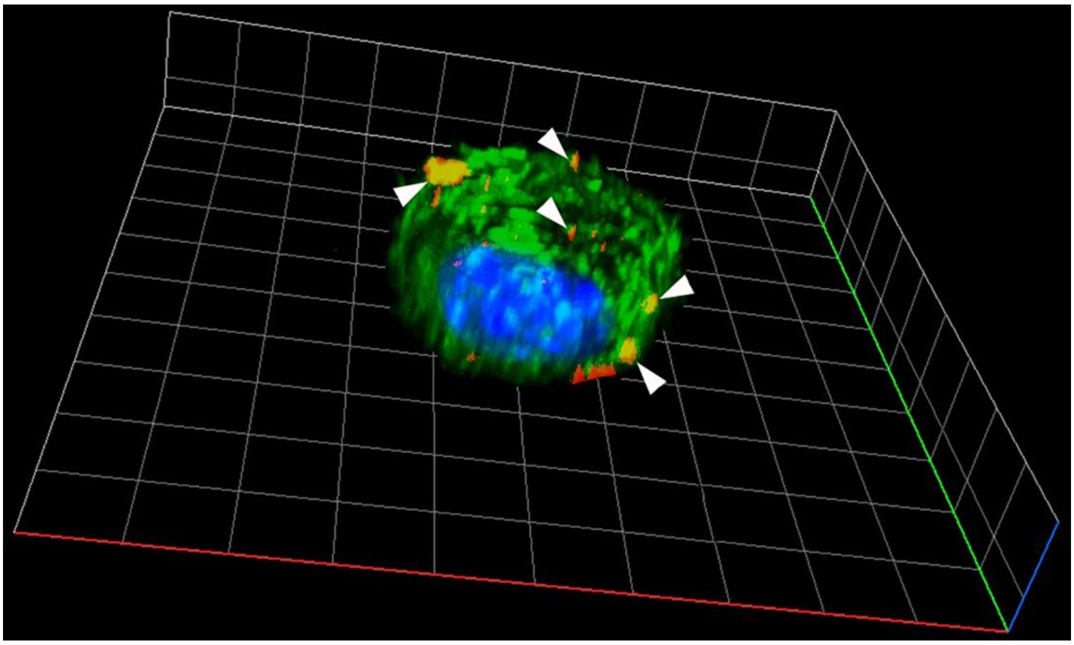

Figure S11. A z-stack of CLSM image for MHV-A59 binding and co-localization to the surface of AM membrane after coincubation for 30 min. The MHV-A59 particles were labeled with quantum dots (red); The AM membrane was stained with DiO dye (green); The AM cell nuclei were stained with DAPI (blue). Orange (white arrowheads) indicated that MHV-A59 co-localized on the surface of AM membrane.

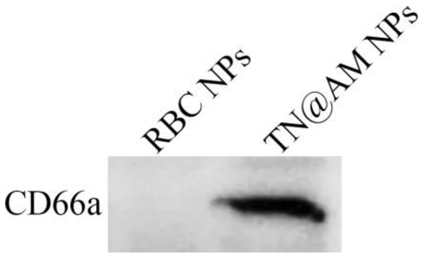

Figure S12. Western blotting analysis for CD66a receptor of RBC NPs and TN@AM NPs. Samples were run at equal protein concentration.

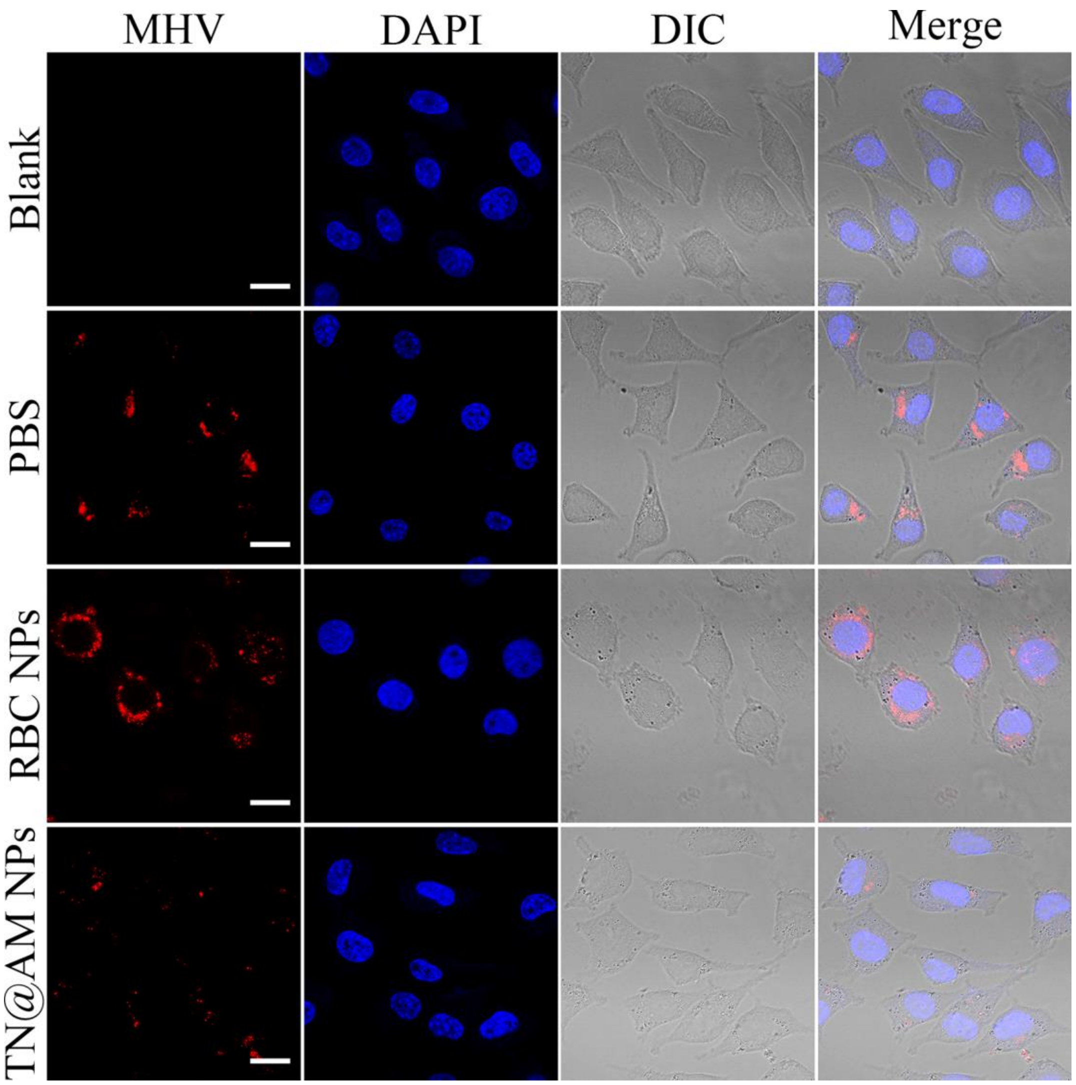

Figure S13. MHV-A59 cellular entry inhibition analysis of PBS, RBC NPs (2.0 mg mL^{-1} protein concentration) and TN@AM NPs (2.0 mg mL^{-1} protein concentration). L929 cells as target cells. The MHV-A59 particles are labeled with quantum dot (red) and L929 cell nuclei are stained with DAPI (blue). Scale bars: 20 μm.

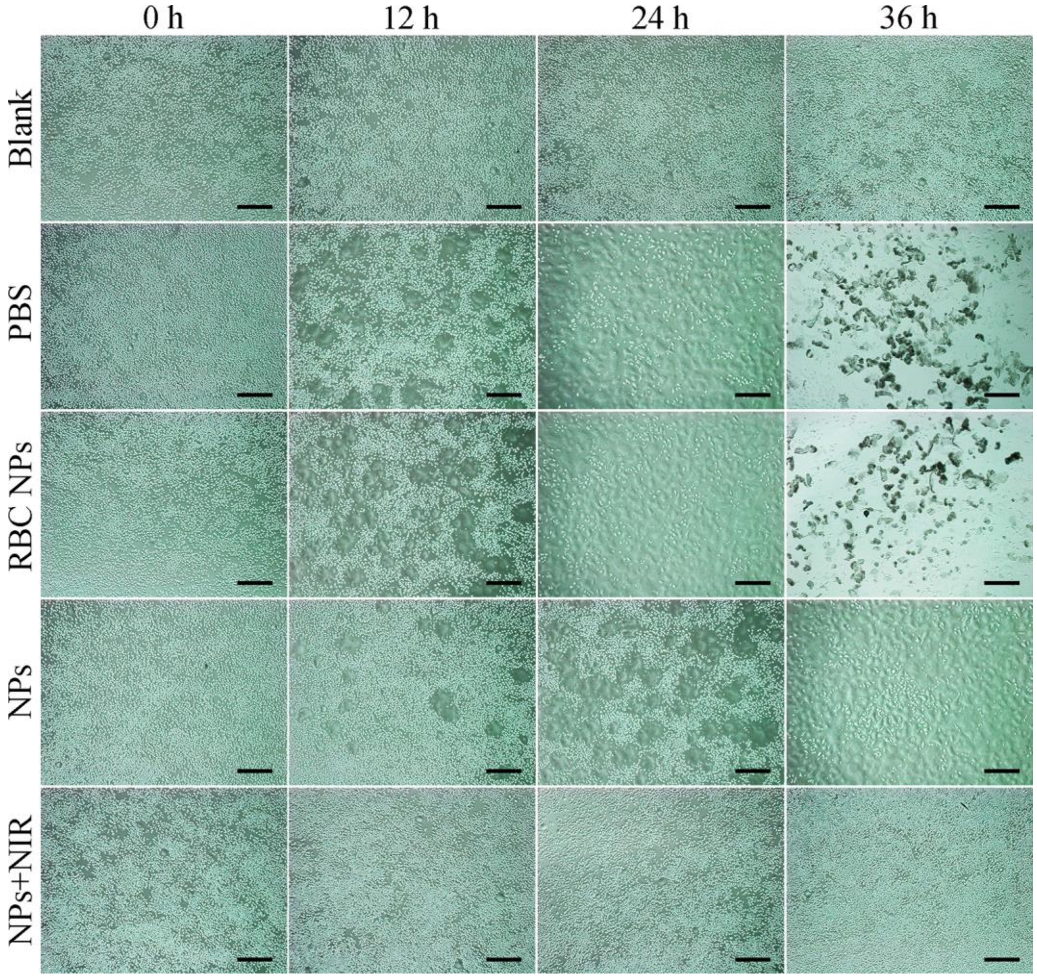

Figure S14. The morphological changes of L929 cells after different treatments followed by virus replication for different times. Scale bars: 200 μm.

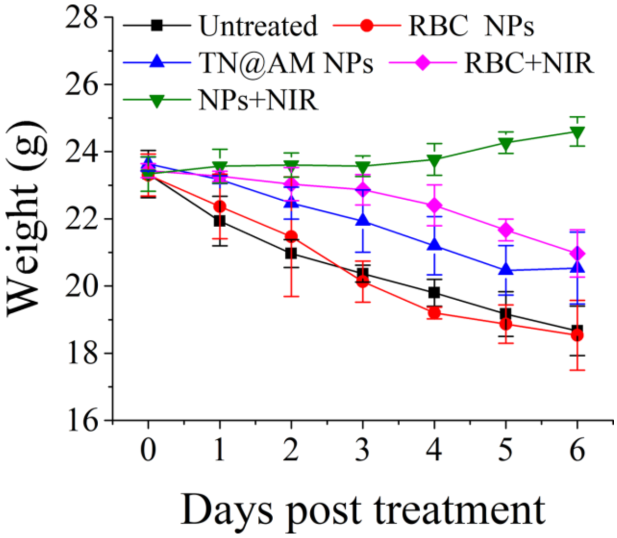

Figure S15. Body weight change analysis of mice after different treatments followed by virus replication for different times.

Table S1. Drug loading content of 2TPE-2NDTA in TN@AM NPs and RBC NPs.

2TPE-2NDTA feeding weight (mg)[a]	EE% in TN@AM NPs[b]	EE% in RBC NPs[b]	Drug loading ratio (%) in TN@AM NPs[c]	Drug loading ratio (%) in RBC NPs[c]
0.20	90.6 ± 9.4	85.8 ± 9.3	1.04 ± 0.12	9.88 ± 0.12
0.40	89.7 ± 7.6	92.5 ± 5.4	2.13 ± 0.18	2.13 ± 0.12
0.60	91.8 ± 5.8	95.1 ± 5.1	3.07 ± 0.17	3.37 ± 0.18
**0.80**	**92.5 ± 4.9**	**93.8 ± 5.8**	**4.16 ± 0.22**	**4.24 ± 0.28**
1.00	74.9 ± 7.4	74.4 ± 9.3	4.06 ± 0.39	4.26 ± 0.50

The data are presented as mean ± SD (n = 3). [a]2TPE-2NDTA were mixed with 10.0 mg PLGA polymer to prepare 2TPE-2NDTA loaded PLGA cores; TN@AM NPs and RBC NPs were prepare at a membrane protein-to-polymer weight ratio of 1:1. [b]Encapsulation efficiencies (EE%) of 2TPE-2NDTA in the NPs were calculated using the formula: (weight of 2TPE-2NDTA loaded)/(feeding weight of 2TPE-2NDTA for NPs preparation)×100. [c]Drug loading ratios (%) of 2TPE-2NDTA in the NPs were calculated using the formula: (weight of 2TPE-2NDTA loaded)/(total weight of TN@AM NPs or RBC NPs after lyophilization) × 100.
